# Comprehensive Integrated Single‐Cell and Spatial Transcriptomics Unveil the Dynamic Landscape of Betel Nut‐Associated Oral Mucosal Carcinogenesis and Its Tumor Microenvironment

**DOI:** 10.1002/mco2.70796

**Published:** 2026-05-31

**Authors:** Wei Dong, Shuojin Huang, Yijun Wu, Congyuan Cao, Jiaxue Li, Qianting He, Anxun Wang

**Affiliations:** ^1^ Department of Oral and Maxillofacial Surgery The First Affiliated Hospital Sun Yat‐Sen University Guangzhou Guangdong China; ^2^ Guangzhou Women and Children's Medical Center Guangzhou Medical University Guangzhou Guangdong China

**Keywords:** antigen‐presenting cancer‐associated fibroblasts, betel nut‐associated oral squamous cell carcinoma, epithelial–mesenchymal transition, LAMC2^+^ epithelial, single‐cell and spatial transcriptomics

## Abstract

Betel nut chewing is a major etiological factor for oral squamous cell carcinoma (OSCC), yet its mechanistic underpinnings remain poorly defined. Here, we performed single‐cell RNA sequencing and spatial transcriptomics from six OSCC patients to comprehensively dissect the tumor microenvironment (TME) dynamics and cellular heterogeneity associated with betel nut‐induced oral mucosal carcinogenesis. We identify a fibrotic, immunosuppressive TME characterized by expanded cancer‐associated fibroblasts (CAFs) and B/plasma cells, alongside depletion of cytotoxic T/NK cells and macrophages. CAFs, particularly antigen‐presenting CAFs, are spatially enriched at the invasive front and drive epithelial plasticity and malignant transformation. Notably, we uncover a malignant epithelial subpopulation, LAMC2^+^ EpiC6, enriched for epithelial–mesenchymal transition (EMT) programs, angiogenesis, and metastasis‐associated pathways, which engaged in extensive crosstalk with CAFs and other nonmalignant components. Clinically, LAMC2 expression was significantly elevated in OSCC tissues from betel nut chewers, and arecoline treatment of OSCC cell lines induced LAMC2 upregulation, EMT, and enhanced migratory and invasive capacities in vitro. Collectively, our study delineates a malignant trajectory of epithelial cell progression, highlighting LAMC2^+^ EpiC6 as a key aggressive subpopulation orchestrated by EMT‐related transcriptional regulators and extracellular matrix remodeling. These findings offer mechanistic insights and identify potential therapeutic targets to disrupt tumor–stroma interplay and mitigate disease progression.

## 1 Introduction

Epidemiological analysis reveals that 120,200 oral cancer cases globally in 2022 were etiologically linked to smokeless tobacco or betel nut use [1]. Betel nut chewing, consumed by approximately 600 million people in the world, is one of the major risk factors for oral cancer and has been evaluated as a Group 1 carcinogen by the International Agency for Research on Cancer. The risk of oral cancer increases in a dose‐response manner with the daily number of betel nuts consumed and the number of years chewing [2]. Arecoline alkaloid present in betel nuts has been reported to be the chief etiological factor causing oral submucous fibrosis (OSF) with a high malignant transformation rate of 7.6% [3]. In China, epidemiologic and clinical studies have reported that the overall malignant transformation rate of OSF is 1.2–2.2% [4]. Numerous studies have investigated the underlying mechanisms by which betel nut chewing contributes to the development of OSF [2,^3^, ^5^, ^6^]. Among genetic studies, a specific association of betel nut use among patients with OSF relates to six collagen‐related genes: COL1A1, COL1A2, collagenase‐1, TGF‐β1, lysyl oxidase, and cystatin C [2]. Hu et al. revealed that overexpression of *DEC1* in the epithelium of OSF induced activation of FAK/Akt signal axis, caused mesenchymal transition, and may promote malignant transformation of OSF [6].

Studies conducted using human, animal, and cellular models have generated evidence of the carcinogenicity of betel nut chewing in humans. Ko et al. clarified that carcinogenesis induced by betel nut chewing comprises the following six steps from cancer initiation and progression: BQ (betel quid)/BN (betel nut) use disorder or addiction; arecoline and arecoline N‐oxide formation in oral cavity; epithelial–mesenchymal transition (EMT) inducers’ activation; ROS formation, cytotoxicity, genotoxicity, and inflammation; genetic and epigenetic pathway dysfunction; and the EMT induced cancer and metastasis [7]. Chang et al. revealed that betel nut components may promote oral squamous cell carcinoma (OSCC) development by causing abnormal cell differentiation, cell damage, and increased COX‐2 and PGE2 levels [8]. Kuo et al. demonstrated that betel nuts promote carcinogenesis through dose‐dependent upregulation of tumor suppressor proteins (p53, NOTCH1, and FAT1) in oral epithelial cells and dysplastic tissues [9].

Cancer initiation and progression are believed to be associated with the tumor microenvironment (TME), which contains various cell types, including fibroblasts, immune cells, neoplastic epithelial cells, endothelial cells, and pericytes. The crosstalk between cancer cells and matrix cells plays a critical role in tumor promotion and suppression [10]. To better understanding the carcinogenesis of oral mucosa and the crosstalk between cancer cells and TME, single‐cell RNA sequencing (scRNA‐seq) and spatial transcriptomics had been used, which can provide an unprecedented opportunity to systematically dissect the cellular heterogeneity and obtain whole‐transcriptome data within tissue sections [11]. Zhang et al. shows that immunosuppressive TME has formed at oral leukoplakia (OLK) stage before transformation into OSCC, as evidenced by increased immunosuppressive cells such as exhausted T cells and some subsets of macrophages and fibroblasts [12]. Hu et al. systematically profiled the dynamic changes in the microenvironment of OSCC and OLK by single‐cell analysis and found that CD4^+^ T cells differentiated into activated Tregs with highly immunosuppressive functions and highly exhausted CD4^+^ T cells and that CD8^+^ T cells consistently converted to an exhausted state. Further analysis showed that a subset of TDO2^+^ myofibroblasts was the key factor contributing to the immunosuppressive microenvironment of OSCC [13].

Recently advances in single‐cell transcriptomic technologies have also provided deeper insights into the molecular alterations driving BN‐associated OSCC. Huang et al. established a mouse model of tongue carcinogenesis using 4‐nitroquinoline 1‐oxide and arecoline, and identified 17 distinct cellular clusters participating in tumor development by scRNA‐seq analysis. Two cell subtypes, subtypes 7 (stem cells) and 9 (keratinocytes), were inferred to be influential oncogenic factors in carcinogenesis. The most significant regulatory pathway involved in the two cell subtypes is MYC_targets_v1 [14]. Through scRNA‐seq, Kurkalang et al. demonstrated that OSF‐associated OSCC tumors exhibit a unique molecular signature. The tumorigenic process appears to be predominantly driven by partial EMT (pEMT) [15].

Despite increasing recognition of the carcinogenic potential of betel nut chewing, the underlying mechanisms driving OSCC in this context remain poorly defined. Here, we integrate single‐cell and spatial transcriptomics to chart the cellular and molecular landscape of betel nut‐associated OSCC. Our findings reveal a fibrotic, immunosuppressive TME, marked by the expansion of cancer‐associated fibroblasts (CAFs) and B/plasma cells and depletion of cytotoxic immune populations. Notably, LAMC2^+^ malignant epithelial cells (EpiC6) emerge as key drivers of tumor progression, engaging in reciprocal interactions with CAFs via collagen– and laminin–integrin pathways. These networks reinforce CAF activation and promote epithelial plasticity, EMT, and metastasis. Importantly, our clinical immunohistochemical analysis further demonstrated significantly elevated LAMC2 expression in OSCC tissues from betel nut chewers, and arecoline treatment of OSCC cell lines recapitulated LAMC2 upregulation, EMT induction, and enhanced migratory and invasive capacities in vitro, providing functional validation of the betel nut–LAMC2–EMT axis. Collectively, our study delineates a malignant trajectory of epithelial transformation in betel nut‐associated OSCC, highlighting LAMC2^+^ EpiC6 as a pivotal subpopulation regulated by EMT‐associated transcription factors (TFs) and matrix interactions. These findings provide a comprehensive framework for understanding the dysregulated intercellular communication networks that underlie OSCC progression in the context of betel nut carcinogenicity, and identify potential therapeutic targets aimed at disrupting tumor–stroma interactions to attenuate disease advancement.

## 2 Results

### 2.1 Dynamic Single‐Cell and Spatial Transcriptomic Landscape of the TME in Betel Nut‐Associated OSCC

**FIGURE 1:**
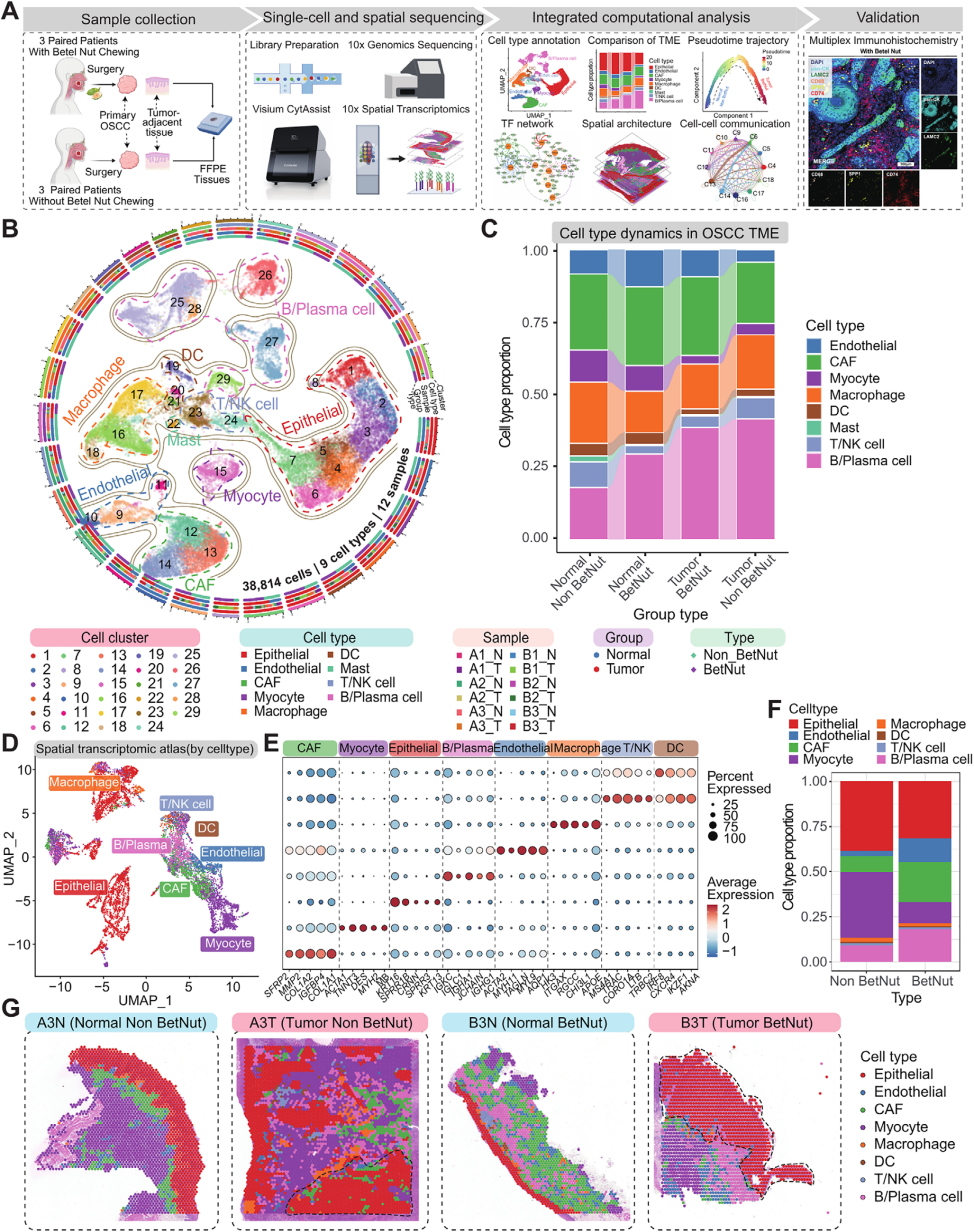
Comprehensive single‐cell and spatial transcriptomic landscape of the TME in betel nut‐associated OSCC. (A) Schematic illustration of the overall study design. Single‐cell and spatial transcriptomic profiling was performed on TME samples derived from betel nut‐associated OSCC to uncover cellular composition, spatial organization, and dynamic gene expression features. Several graphical elements in this figure were created using BioRender (https://www.biorender.com/). (B) Circular UMAP projection of integrated single‐cell transcriptomes (*n* = 38,814) from 12 OSCC patient samples. Each dot denotes an individual cell, with colors representing distinct cell clusters. Colored irregulars delineate nine major cell types. Five colored tracks (from outside to inside) indicate cell clusters, cell types, sample ID, sample groups, and sample types. (C) Stacked bar plot showing the cell proportion dynamics of major cell types across four different sample group types. Colors represent different cell types. (D) UMAP visualization of spatial transcriptomic spots derived from four samples in betel nut‐associated OSCC. Colors represent different cell types. (E) Bubble plots showing the relative average expression of top 5 marker genes (*x*‐axis) for each major cell type (*y*‐axis). Dot size represents the proportion of cells expressing the gene within each cell type, and color intensity reflects relative average gene expression. (F) Stacked bar plot showing the cell proportion of major cell types between non‐betel nut and betel nut groups. Colors represent different cell types. (G) Spatial plots depicting the distribution of annotated cell types across different samples. Colors represent different cell types, with tumor regions marked by irregular outlines.

To unravel the molecular and cellular mechanisms underlying the malignant progression of OSCC in the context of habitual betel nut chewing, we performed integrative scRNA‐seq and spatial transcriptomics to comprehensively dissect the TME dynamics and cellular heterogeneity in paired tumor and adjacent histologically normal oral mucosa from six OSCC patients (Figure 1A), three with betel nut chewing and three without (Table S1). Following stringent quality control and removal of low‐quality cells and sparsely expressed genes, we retained overall gene expression profiles from 38,814 high‐quality single cells. These included 14,168 cells from the betel nut chewing group (10,899 tumor‐derived, 3269 normal‐adjacent) and 24,646 cells from the nonchewing group (12,953 tumor‐derived, 11,693 normal‐adjacent). To mitigate potential batch effects and integrate multiple data across samples, we applied the Seurat implemented canonical correlation analysis (CCA) algorithm, followed by Uniform Manifold Approximation and Projection (UMAP)‐based nonlinear dimensionality reduction to visualize cell clustering (Figures 1B and S1A–D).

Cell type annotation was subsequently conducted based on canonical marker expression and cluster‐specific differentially expressed genes (DEGs) (Figure S1E and Table S2), identifying nine major cellular lineages within the dataset. These included: tumor epithelial cells (*KRT5* and *KRT14*), endothelial cells (*PECAM1* and *VWF*), CAFs (*COL3A1* and *DCN*), myocytes (*ACTA1* and *MYL1*), macrophages (*C1QA* and *APOE*), dendritic cells (DCs; *IRF8* and *CLEC4C*), mast cells (*CPA3* and *KIT*), B/plasma cells (*CD79A* and *IGKC*), and T/NK lymphocytes (*CD3D* and *NKG7*) (Figure 1B). Strikingly, comparative analysis revealed profound shifts in cellular composition between the betel nut chewing and nonchewing groups. Specifically, the stromal cells (CAF and endothelial) and B/plasma cells infiltration was elevated from nonchewing mucosa to betel nut‐associated mucosa and carcinoma, whereas the macrophage and T/NK cell proportions exhibited a modest decline (Figures 1C and S1F). Together, these findings delineate a dynamic stromal and immunological landscape in betel nut‐associated OSCC characterized by CAF and B/plasma cell expansion, accompanied by diminished macrophage and cytotoxic T/NK lymphocyte presence, consistent with an immunosuppressive and fibrotic microenvironment conducive to tumor progression in bet nut‐associated OSCC TME.

To further elucidate the spatial architecture and heterogeneity of cellular compartments, we conducted spatial transcriptomic profiling on four representative samples, encompassing tumor and adjacent normal tissues from both betel nut chewing and nonchewing patients (Table S1). Integration of spatial transcriptomic data with corresponding scRNA‐seq‐derived annotations enabled robust mapping of cell identities, resulting in a comprehensive high‐quality spatial transcriptomic atlas (*n* = 10,559 spots) of the TME in betel nut‐associated OSCC (Figures 1D,E and S1G–I and Table S3). Intriguingly, spatial transcriptomics corroborated key trends observed in the single‐cell data, reinforcing the elevated presence of stromal cells (CAF and endothelial) and B/plasma cells alongside reduced macrophages and T/NK cell infiltration in betel nut users (Figure 1F). Additionally, a pronounced accumulation of CAFs was observed in the betel nut‐associated mucosal context, underscoring a tumor‐supportive stromal remodeling that may actively contribute to OSCC progression. Moreover, we uncovered a notable spatial patterning that macrophages and CAFs preferentially localized at the invasive fronts and periphery of tumor epithelial cells in betel nut‐associated tumors (Figure 1G).

### 2.2 Identification and Characterization of a Highly Invasive LAMC2^+^ Malignant Epithelial Subpopulation in Betel Nut‐Associated OSCC

**FIGURE 2:**
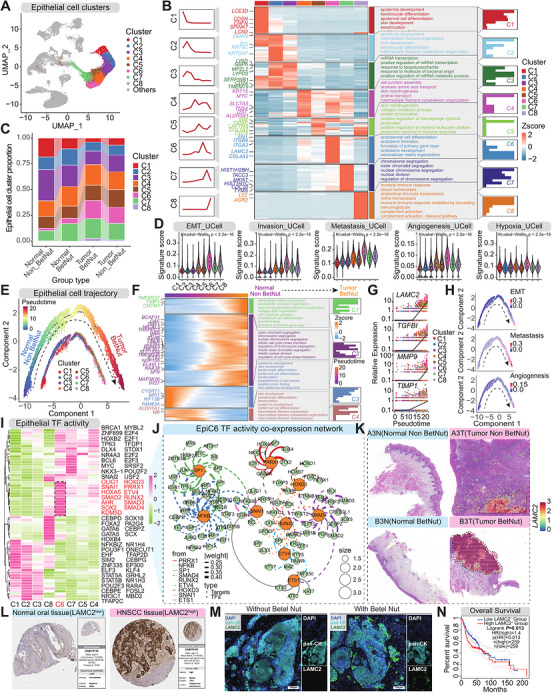
Identification and characterization of a highly invasive LAMC2^+^ malignant epithelial subpopulation in betel nut‐associated OSCC. (A) UMAP plot showing the eight epithelial cell subclusters in betel nut‐associated OSCC. Colors represent different cell clusters. (B) Heatmap of cell cluster‐specific marker gene expression patterns. The left panel features smoothed average expression trends across clusters, while the right panel annotates enriched GO terms associated with each cluster. (C) Stacked bar plot showing the cell proportion dynamics of eight epithelial cell subclusters across four different sample group types. Colors represent different cell clusters. (D) Violin plots displaying the UCell signature scores for multiple tumor‐associated gene signatures across the eight epithelial subclusters. (E) Pseudotime trajectory plots showing the inferred differentiation trajectory among epithelial cell subclusters, colored by pseudotime (outer) and cell clusters (inner). (F) Pseudotime heatmap showing the differentially expressed gene patterns along the epithelial cell trajectory, with annotated GO enrichment terms in the right. (G) Pseudotime scatter plots showing the dynamic expression of representative genes across the inferred trajectory. Colors represent different cell clusters. (H) Pseudotime projection of tumor‐associated gene signature activity scores along the epithelial cell differentiation. (I) Clustered heatmap showing the average TF activity across distinct epithelial cell subclusters. The LAMC2^+^ C6 epithelial cluster TFs are outlined in black. (J) Coexpression TF network of the LAMC2^+^ C6 epithelial cluster TFs. (K) Spatial plots showing the *LAMC2* gene expression patterns across different samples, with tumor regions of high expression highlighted by irregular outlines. (L) Representative immunohistochemistry images comparing LAMC2 protein levels in normal oral mucosa and HNSCC tissue. (M) Multiplex immunohistochemistry (mIHC) staining showing the spatial distribution of LAMC2^+^ epithelial cells in OSCC tissues from betel nut chewers versus nonchewers. (N) Kaplan–Meier survival plot showing the worse overall survival outcomes in the LAMC2 high‐expression group (log‐rank test, *p* < 0.05) in HNSCC patients.

To further unravel the heterogeneity and dynamic evolution of tumor epithelial cells within the TME of betel nut‐associated oral cancers, we performed an in‐depth comparative analysis of the eight epithelial subclusters, designated as EpiC1 (*KRT78*, *CRCT1*, and *IL36A*), EpiC2 (*KRT6C*, *KRT16*, and *KRTDAP*), EpiC3 (*KRT4*, *KRT13*, and *AQP3*), EpiC4 (*KRT15*, *CXCL14*, and *COL17A1*), EpiC5 (*LAPTM5*, *COL1A2*, and *TXNIP*), EpiC6 (*LAMC2*, *MMP9*, and *SERPINE1*), EpiC7 (*HIST1H1B*, *HIST1H1C*, and *TOP2A*), and EpiC8 (*MUC5B*, *LTF*, and *BPIFB1*) (Figures 2A and S2A). Each subpopulation exhibited distinct transcriptional signatures (Figure S2A and Table S4), suggestive of divergent functional roles. Gene Ontology (GO) enrichment analysis further underscored the functional divergence among these epithelial subclusters. For example, EpiC1 cells were primarily enriched in pathways related to epidermis development, keratinocyte differentiation, and epidermal cell differentiation. The highly proliferative EpiC7 cluster was significantly enriched in biological processes including chromosome segregation and mitotic sister chromatid segregation. Notably, the EpiC6 subpopulation exhibited substantial enrichment in pathways associated with endoderm cell differentiation, endoderm formation, and extracellular matrix organization, underscoring its potential role in promoting invasive behavior and tumor progression (Figure 2B). These data collectively highlight a high degree of epithelial cell heterogeneity in oral cancers, with distinct subpopulations potentially contributing specialized roles in tumor progression.

Comparative analysis of subpopulation proportions revealed a marked shift in epithelial cell composition in betel nut‐associated OSCC. Specifically, the relative abundance of EpiC6 and EpiC7 subclusters was greatly increased, whereas EpiC1 and EpiC3 subclusters were diminished from normal mucosa to betel nut‐associated mucosa and tumors (Figures 2C and S2B). This suggests that EpiC6 and EpiC7 cells may represent key drivers of malignant progression in the context of betel nut exposure, characterized by heightened proliferation and invasive potential. Supporting this, tumor‐associated gene signature (Table S5) enrichment analysis revealed a pronounced activation of EMT programs, angiogenesis, invasion, and metastasis‐associated pathways specifically within the EpiC6 subpopulation (Figures 2D and S2C), further implicating these cells as highly malignant drivers in betel nut‐associated OSCC.

To further delineate the developmental trajectories and dynamic plasticity of these epithelial subpopulations, we employed pseudotime trajectory analysis using Monocle2 [16, 17]. The reconstructed trajectories unveiled a clear lineage progression, wherein EpiC1 cells occupied the initial branchpoint of the trajectory, while the EpiC6 cluster, notably marked by high *LAMC2* expression, localized at the terminal ends of the trajectory (Figures 2E and S2D,E). This indicates that EpiC6 cells represent terminally differentiated, highly malignant tumor epithelial cells in the context of betel nut‐induced carcinogenesis. Complementary GO enrichment analysis of pseudotime‐dependent DEGs revealed significant enrichment of extracellular matrix organization, extracellular structure organization, and collagen fibril organization pathways during the transition from EpiC1 to EpiC6 cells (Figure 2F–H). Together, these findings implicate extracellular matrix remodeling and EMT‐related processes as key biological events underlying betel nut‐associated malignant transformation.

To uncover potential regulatory mechanisms underpinning epithelial plasticity and malignant differentiation, we performed TF activity enrichment and coexpression regulatory network analysis across epithelial subpopulations. Distinct TF modules were found to orchestrate the transcriptional programs of each subpopulation (Figure 2I). Strikingly, the EpiC6 subpopulation exhibited elevated activity of EMT‐ and invasion‐associated TFs, including SNAI1, RUNX2, SMAD4, and PRRX1, suggesting that these factors serve as pivotal regulators driving the betel nut‐associated malignant transformation and invasive capacity of EpiC6 cells (Figure 2J). Furthermore, spatial transcriptomic analysis corroborated the single‐cell findings by revealing a pronounced enrichment of LAMC2^+^ malignant epithelial cells within tumor tissues in betel nut chewers, with a spatial preference toward the tumor core and invasive front (Figures 2K and S2F). To further validate the expression and functional significance of *LAMC2* in OSCC, we examined immunohistochemistry (IHC) data for HNSCC from the Human Protein Atlas (https://www.proteinatlas.org/) database. LAMC2 protein levels were markedly elevated in tumor tissues compared with normal oral mucosa (Figure 2L). Concordantly, our spatial transcriptomic data confirmed that *LAMC2* expression was highest in OSCC samples from betel nut chewers (Figure S2G). Multiplex IHC (mIHC) further substantiated these findings, showing a significantly higher proportion of LAMC2^+^ malignant epithelial cells in the tumor tissues of betel nut chewers relative to nonchewers (Figures 2M and S2H). Moreover, survival analysis using the GEPIA2 [18] website revealed that high *LAMC2* expression was associated with significantly poorer overall prognosis in HNSCC patients (Figure 2N), suggesting that *LAMC2* not only serves as a marker of aggressive tumor phenotypes but may also represent a potential prognostic indicator and therapeutic target in betel nut‐related OSCC.

Collectively, these findings delineate a malignant trajectory of tumor epithelial cell progression, highlighting LAMC2^+^ EpiC6 as a key aggressive subpopulation orchestrated by EMT‐related transcriptional regulators and extracellular matrix remodeling, thus providing potential therapeutic targets for mitigating betel nut‐induced oral cancer progression.

### 2.3 Dissecting the Heterogeneity and Functional Dynamics of Stromal Cells in the TME of Betel Nut‐Associated Oral Cancer

**FIGURE 3:**
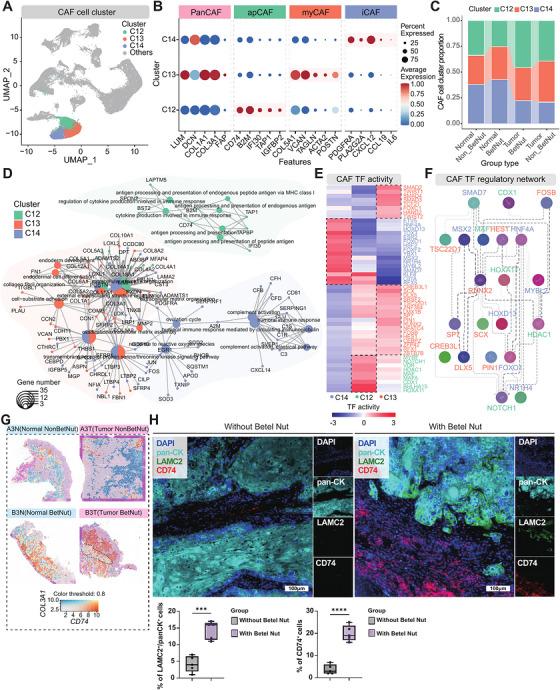
Dissecting the heterogeneity and functional dynamics of CAF cells in the TME of betel nut‐associated oral cancer. (A) UMAP embedding of CAFs identifies three transcriptionally distinct CAF subpopulations in betel nut‐associated OSCC. Each dot represents an individual cell, and colors denote the identified subclusters. (B) Bubble plots showing the relative average expression of representative marker genes (*x*‐axis) across the three CAF cell subtypes (*y*‐axis). Dot size denotes the percentage of cells in a cell type expressing the gene. Dot color represents the relative average expression level. (C) Stacked bar plot depicting the proportional distribution of the three CAF subclusters across four different sample group types. Colors represent different cell clusters. (D) Functional enrichment network of GO terms and associated genes across different CAF cell subclusters. Colors represent different cell clusters. (E) Clustered heatmap showing the average TF activity scores in each CAF cell subclusters. Each cluster TFs were highlighted with black border. (F) Regulatory network of TFs in CAF subclusters. Colored TFs indicate CAF subclusters, corresponding to Figure 3E. (G) Spatial transcriptomic plots showing the coexpression patterns of *COL3A1* and *CD74* genes across different samples, with CD74^+^ apCAF marked by irregular outlines. (H) Representative mIHC images demonstrating the spatial distribution of LAMC2*^+^* epithelial cells and CD74*^+^* apCAFs in oral tumor tissues from betel nut chewers and nonchewers. Box plots (bottom) quantify the relative abundance of LAMC2*^+^* epithelial and CD74*^+^* apCAF cells between the two patient groups (*n* = 5, ***p < 0.001, ****p < 0.0001 by Student's *t*‐test).

To further elucidate the heterogeneity and dynamic reprogramming of stromal cells within the TME of betel nut‐associated OSCC, we performed a detailed comparative analysis focusing on CAF subpopulations and endothelial cells (Figures 3A and S3A). Based on the expression patterns of well‐established CAF marker genes, CAF populations were categorized into three distinct subtypes: antigen‐presenting CAF (apCAF) (C12), characterized by high expression of *CD74*, *B2M*, and *TAP1*; myCAF (C13), marked by *COL5A1*, *VCAN*, and *TAGLN* expressions; iCAF (C14), defined by elevated levels of *PDGFRA*, *CXCL12*, and *CCL19* (Figure 3B and Table S6). Cell proportion comparative analysis revealed a notable expansion of apCAF or iCAF populations in the betel nut‐associated tumor or normal samples respectively (Figure 3C), suggesting their distinct crucial involvement in driving tumor progression and inflammatory progression for remodeling the TME to favor malignancy. GO enrichment analysis provided functional insights into each CAF subtype. apCAFs showed significant enrichment in pathways including antigen processing and presentation via MHC Class I, extracellular structure organization, collagen fibril organization, and regulation of cytokine production involved in immune response, indicating dual roles in both matrix remodeling and modulating immune responses. myCAFs were strongly enriched in extracellular matrix organization, collagen fibril organization, cell‐substrate adhesion, and endodermal cell differentiation, reflecting their prototypical function in matrix deposition, tissue stiffness regulation, and promotion of an invasive tumor phenotype. iCAFs displayed enrichment in complement activation, humoral immune response, ECM organization, and regulation of complement activation, suggesting they act as key players in orchestrating immune responses and mediating inflammatory crosstalk within the TME (Figure 3D). These findings collectively underscore the extensive functional heterogeneity of CAF populations and highlight their distinct yet complementary roles in shaping the protumorigenic niche.

To uncover the transcriptional regulatory mechanisms underlying the differentiation and functional specialization of CAF subpopulations, we performed TF activity enrichment analysis. In apCAFs, TFs such as HOXA11, BHLHA15, HDAC1, NOTCH1, and MAF exhibited heightened activity, suggesting their involvement in regulating antigen presentation machinery and immune modulatory pathways. myCAFs were predominantly regulated by TFs including SMAD2, SMAD4, PIN1, RUNX2, and TCF21, consistent with their role in TGF‐β signaling‐driven matrix remodeling, contractility, and myofibroblastic differentiation. In iCAFs, TFs such as CIITA, RFX5, ETV5, and FOXO1 showed prominent activity, reflecting their capacity to regulate immune‐associated gene expression and inflammatory signaling cascades (Figure 3E,F). These TF networks suggest that each CAF subtype is governed by distinct transcriptional programs, orchestrating their specialized contributions to tumor progression, immune evasion, and extracellular matrix remodeling. Furthermore, spatial transcriptomic profiling revealed a marked enrichment of CD74^+^ apCAFs within tumor tissues of betel nut chewers, with a predominant localization at the invasive tumor margins (Figure 3G). To validate the presence and spatial distribution of apCAFs in OSCC, we performed mIHC. Consistent with the transcriptomic data, CD74^+^ apCAFs were significantly more abundant in tumors from betel nut chewers compared with nonchewers, and were primarily concentrated at the interface between stromal regions and LAMC2^+^ malignant epithelial cells (Figure 3H). These findings highlight a spatially confined expansion of apCAFs in betel nut‐associated OSCC, potentially contributing to tumor–stroma crosstalk at the invasive front.

Endothelial cells were similarly dissected and categorized into two major categories based on canonical marker gene expression: blood vascular endothelial cells (C9 and C10, characterized by high expression of *VWF*, *IGFBP3*, *ACKR1*) and lymphatic endothelial cells (C11, marked by *PDPN*, *CCL21*, *PROX1*) (Figure S3B and Table S7). Further subclassification of blood vascular endothelial cells revealed distinct arterial (C9) and venous (C10) endothelial subsets. Cellular composition analysis demonstrated a marked expansion of the lymphatic endothelial population in the betel nut‐associated OSCC group (Figure S3C), implicating this subpopulation in promoting tumor progression and potentially facilitating lymphatic metastasis. Functional enrichment analysis via GO revealed divergent biological roles among endothelial subsets (Figure S3D). Of particular interest, lymphatic endothelial cells (C11) exhibited enrichment in positive regulation of cell adhesion, promotion of epithelial cell migration, and ECM organization, underscoring their prometastatic potential (Figure S3D). Moreover, this population displayed elevated expression of genes associated with tumor vascularization (*FLT4*, *KDR*, *NRP2*, *TIE1*), vascular morphogenesis (*HES1*, *EFNB3*, *EPHB4*), inflammatory response (*CCL2*, *STAB1*, *NT5E*), and guidance receptors (*ROBO4*, *ROBO3*, *UNC5B*) (Figure S3E), collectively supporting a pivotal role in facilitating angiogenesis, lymphangiogenesis, and tumor dissemination in the context of betel nut‐associated OSCC.

Taken together, our comprehensive interrogation of stromal cell heterogeneity reveals that both the expansion of lymphatic endothelial cells and specific CAF subpopulations, particularly apCAF, play central roles in shaping a protumorigenic microenvironment in betel nut‐associated OSCC. The identified transcriptional regulators and enriched pathways provide novel insights into the mechanisms driving stromal cell‐mediated tumor progression and offer potential therapeutic targets aimed at disrupting the malignant crosstalk within the tumor stroma.

### 2.4 Unrevealing Immune Cell Heterogeneity and Functional Diversity in the Tumor Immune Microenvironment of Betel Nut‐Associated OSCC

**FIGURE 4:**
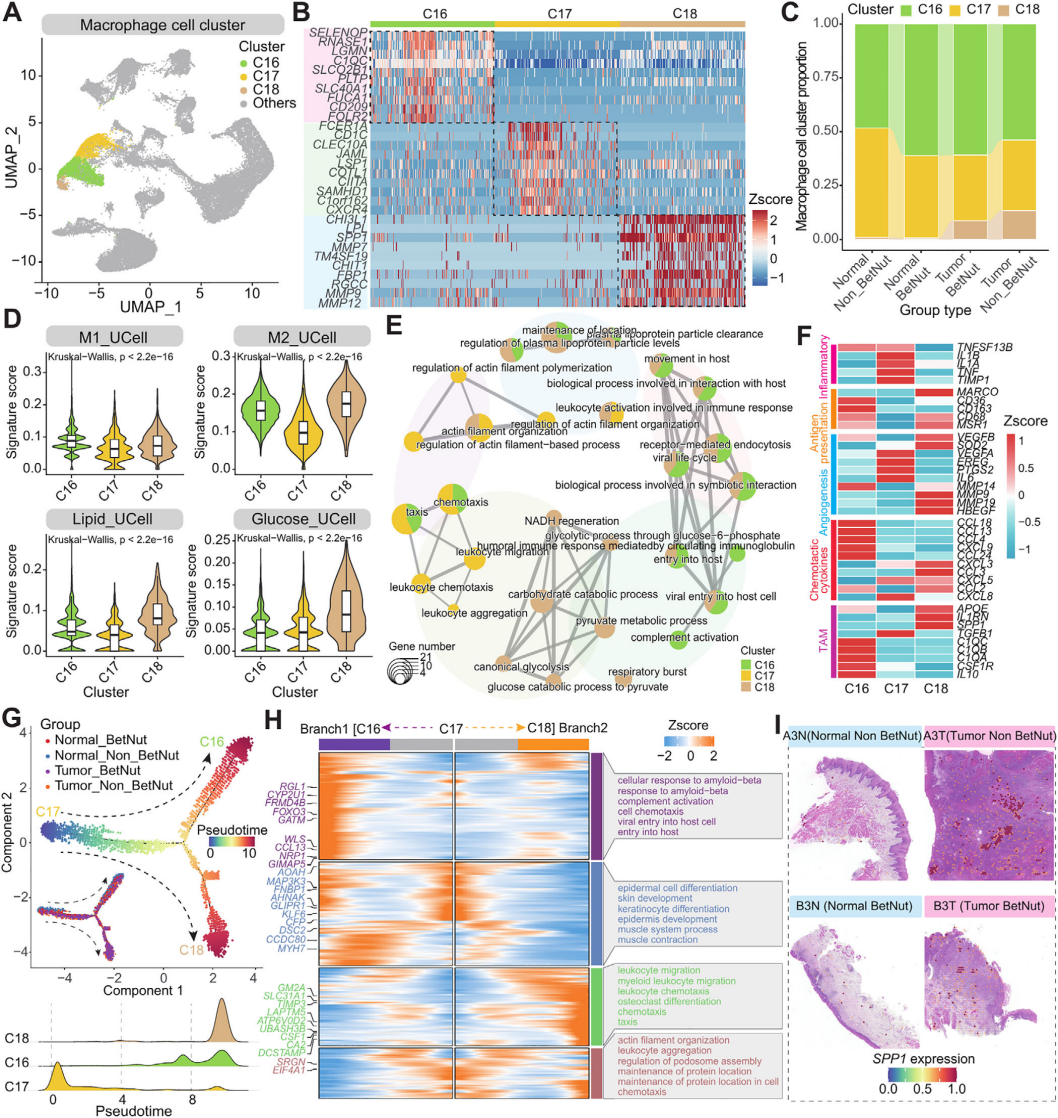
Profiling macrophage heterogeneity and functional diversity in the TME of betel nut‐associated OSCC. (A) UMAP visualization of tumor‐infiltrating macrophages identifies three transcriptionally distinct subclusters in betel nut‐associated OSCC. Each cluster is color coded. (B) Heatmap showing expression profiles of representative DEGs across the three macrophage subclusters. (C) Stacked bar plot illustrating the distribution of macrophage subpopulations across four different sample group types. Colors represent different cell clusters. (D) Violin plots showing the UCell signature scores for macrophage‐related and metabolic gene programs across the three macrophage subclusters. (E) Circular GO term map illustrating enriched biological processes in each macrophage subclusters. Node color indicates cluster origin, and spatial arrangement reflects semantic similarity between terms. (F) Heatmap depicting the expression of key genes associated with immune‐related functional programs across macrophage subclusters. (G) Pseudotime trajectory (top) and ridge plots (bottom) showing the inferred differentiation trajectory among macrophage cell subclusters. (H) Branched pseudotime heatmap showing temporally ordered gene expression changes along divergent macrophage trajectories. Annotated GO terms (right) indicate functionally enriched gene modules associated with each branch. (I) Spatial transcriptomic maps displaying the expression pattern of *SPP1* across tumor tissues in different samples.

The tumor immune microenvironment (TIME) is a highly intricate and dynamic ecosystem of cellular interactions that profoundly shapes tumor evolution, immune evasion, and therapeutic outcomes. Myeloid cells, particularly macrophages and DCs, play a pivotal role in orchestrating immune homeostasis and inflammatory signaling within the TIME. To gain deeper insights into the heterogeneity and dynamic changes of myeloid cells within the TIME of betel nut‐associated oral cancer, we conducted a comparative analysis of macrophages and DCs (Figures 4A and S4A). We classified macrophages into three distinct subpopulations based on cluster‐specific gene expressions, including C16 (*C1QC*, *SELENOP*, and *FOLR2*), C17 (*COTL1*, *LSP1*, and *SAMHD1*), and C18 (*SPP1*, *MMP7*, and *MMP9*) (Figure 4B and Table S8). Comparative abundance analysis revealed a markable increase in C18 macrophages in tumor tissues compared with adjacent normal tissues (Figure 4C). Gene signature enrichment analysis revealed that the C18 subpopulation, characterized by high *SPP1* expression, exhibited the highest M2‐like polarization, and displayed enhanced glucose and lipid metabolic activity, suggesting its pivotal role in immune suppression, metabolic reprogramming, and tumor progression (Figure 4D and Table S9). GO enrichment analysis further indicated that C16 macrophages were predominantly involved in complement activation, receptor‐mediated endocytosis, and humoral immune responses, whereas C17 macrophages were enriched in pathways related to actin filament organization, chemotaxis, and leukocyte migration. Notably, C18 macrophages were significantly enriched in pathways related to glycolysis (NADH regeneration, canonical glycolysis, glucose catabolic process to pyruvate), highlighting their metabolic adaptation in the TIME (Figure 4E). In addition, we identified that SPP1^+^ C18 macrophages highly expressed multiple proangiogenic factors (*MMP9*, *MMP19*, *HBEGF*, *VEGFB*, *SOD2*) and chemokines (*CXCL3*, *CXCL5*, *CCL3*, *CCL2*) (Figure 4F), further underscoring their potential roles in tumor angiogenesis, immune cell recruitment, and tumor progression.

To further investigate the differentiation relationships among macrophage subpopulations, we performed pseudotime trajectory analysis. Our findings revealed that C17 macrophages occupied the early branchpoint of the trajectory, while C16 and C18 macrophages emerged as terminally differentiated populations along distinct pathways, suggesting their divergent functional fates (Figure 4G). Differential trajectory analysis showed that C16 macrophage differentiation was enriched in pathways such as cellular response to amyloid‐beta, complement activation, and viral entry into host cells, reflecting an immune‐modulatory phenotype. In contrast, C18 macrophage exhibited a distinct trajectory primarily associated with leukocyte migration, myeloid leukocyte migration, and osteoclast differentiation, further supporting their immunosuppressive and protumorigenic roles (Figure 4H). In addition, our spatial transcriptomic analysis demonstrated elevated expression of *SPP1* in tumor regions compared with adjacent normal tissue (Figure 4I). This spatial enrichment of *SPP1^+^* macrophages in the TME implicates this subpopulation potentially in promoting OSCC progression. Collectively, these results reveal functionally divergent macrophage trajectories in betel nut‐associated OSCC and position *SPP1^+^* C18 macrophages as key contributors to tumor‐promoting and tissue remodeling.

Similarly, our comparative analysis of DCs classified them into three major subtypes based on canonical marker gene expression: conventional DC1 (cDC1, C19) (*XCR1, CLEC9A*, and *BATF3*), plasmacytoid DCs (pDCs, C20) (*CLEC4C, PLD4*, and *PTGDS*) and LAMP3^+^ DCs (C21) (*LAMP3, FSCN1*, and *CCR7*) (Figure S4B and Table S10). Cellular proportion analysis revealed a notable expansion of pDC populations in betel nut‐associated oral cancer than in betel nut normal mucosa (Figure S4C). Functional enrichment analysis demonstrated that pDC cells were enriched in pathways related to cytokine production, immune effector regulation, and TNF superfamily cytokine production, underscoring their involvement in inflammatory responses (Figure S4D).

In addition to myeloid alterations, we observed a pronounced enrichment of B/plasma cells within the TIME of betel nut‐associated OSCC. Comparative analyses revealed that these B lineage cells exhibit substantial heterogeneity, allowing us to classify them into five distinct subpopulations, including C25 (*IGHG1* and *CTSB*), C26 (*IGKC* and *IGHG3*), C27 (*IGHA1* and *JCHAIN*)、C28 (*TOP2A* and *MKI67*), and C29 (Plasma, *MZB1* and *XBP1*) (Figure S4E,F and Table S11). Cellular composition analysis revealed distinct distribution patterns across disease states. Notably, the C25 subpopulation (B_IGHG1) was greatly expanded in betel nut‐associated tumor tissues, whereas the fraction of C29 plasma cells is diminished, pointing to a potential disruption in terminal B cell differentiation (Figure S4G). These shifts highlight a possible functional reprogramming of B lineage cells during malignant transformation and underscore their potential contribution to the immunopathology of betel nut‐associated OSCC. GO enrichment analyses further support these functional distinctions. The C25 subpopulation is primarily enriched in pathways related to complement‐dependent cytotoxicity, acute inflammatory responses to antigenic challenge, activation of the classical complement cascade, and collagen catabolism (Figure S4H). Together, these dynamic alterations in the composition and functional state of B/plasma cell subsets underscore their critical involvement in the malignant transformation and progression of betel nut‐associated oral carcinoma.

In summary, our study systematically delineates the heterogeneity and functional divergence of myeloid and B lineage cells in the TIME of betel nut‐associated oral cancer, highlighting distinct macrophage and DC subsets with immunosuppressive, proangiogenic, and metabolic adaptations. The identification of metabolically reprogrammed, immunosuppressive SPP1^+^ C18 macrophages, and the expansion of proinflammatory B_IGHG1 (C25) cells highlights key cellular players driving immune dysregulation and tumor progression in this etiologically distinct cancer subtype. These findings collectively offer a refined framework for understanding myeloid‐driven immune dysregulation and provide potential targets for precision immunotherapy in oral cancer.

### 2.5 Cell–Cell Interaction Reveals Tumor Epithelium–CAF Crosstalk Driving Malignant Transformation and Invasive Progression in Betel Nut‐Associated OSCC

**FIGURE 5:**
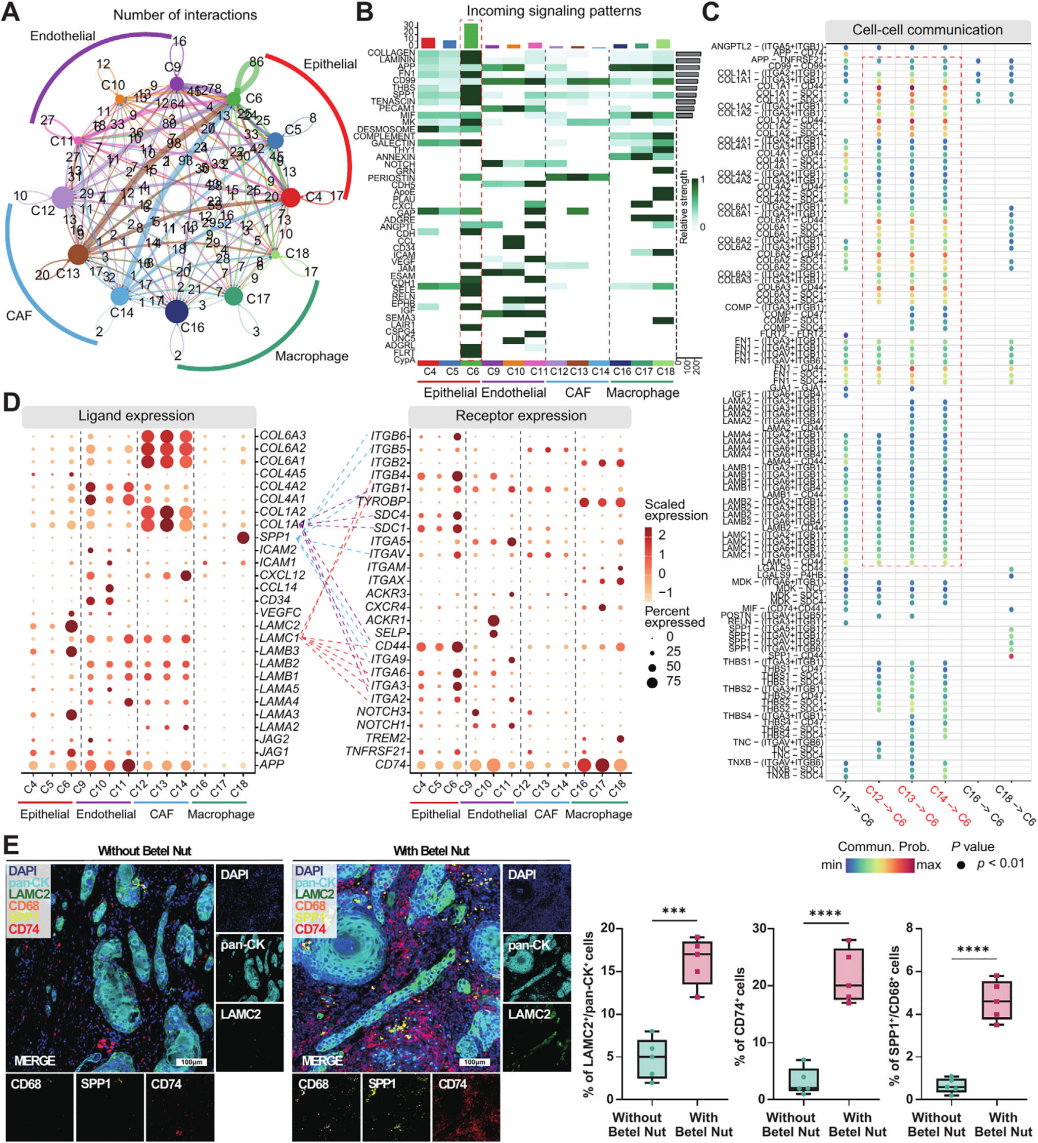
Malignant epithelium–CAF crosstalk drives tumor progression in betel nut‐associated OSCC. (A) Circular cell–cell communication network illustrating the number of inferred ligand–receptor interactions among epithelial cells, endothelial cells, CAFs, and macrophages. Node color represents cell type, and edge thickness denotes interaction count. (B) Heatmap showing the strength of incoming signaling pathways among different cell clusters. Each row corresponds to a specific signaling pathway, and columns represent recipient clusters. Cell type identities are annotated below. (C) Bubble plot summarizing the strength of selected ligand–receptor interaction pairs between cell populations. Dot size indicates communication intensity, and color reflects interaction specificity. (D) Bubble plot showing the expression levels of ligands and receptors involved in key signaling axes across different cell subclusters. Dot size indicates the percentage of expressing cells, and color reflects mean expression levels. (E) Representative mIHC images showing the spatial colocalization of LAMC2*^+^* epithelial cells, CD74*^+^* apCAFs, and SPP1^+^CD68*^+^* macrophages in OSCC tissues from betel nut chewers and nonchewers. The corresponding box plots (right) quantify the abundance of each cell population, demonstrating increased infiltration and spatial association in betel nut‐exposed tumors (*n* = 5, ***p < 0.001, ****p < 0.0001 by Student's *t*‐test).

OSCC induced by chronic betel nut exposure presents with a highly dynamic and heterogenous TME, where reciprocal intercellular signaling networks critically shape mucosal malignant transformation, immune evasion, and invasive behavior. To systematically dissect these complex communication networks and uncover their mechanistic underpinnings, we implemented the CellChat [19] computational framework to map the cell–cell signaling landscape across distinct cellular subpopulations within the TME. Our analysis revealed a highly interactive network between malignant epithelial cells, CAFs, endothelial cells, and macrophages, underscoring their pivotal roles in shaping the tumor ecosystem (Figure 5A). Notably, the EpiC6 epithelial subpopulation emerged as a key hub of intercellular communication, exhibiting a broad spectrum of incoming interactions with stromal and immune subsets (Figure 5B). This finding suggests that EpiC6 cells actively engage in reciprocal interactions with CAFs and other nonmalignant components, potentially orchestrating a protumorigenic niche that fosters malignant transformation and disease progression in betel nut‐induced oral mucosal carcinogenesis.

Comparative analysis between tumor and adjacent normal tissues revealed a marked increase in both the number and strength of intercellular signaling events within tumor regions (Figure S5A–C). Several tumor‐enriched signaling pathways, including SPP1, SELE, SELL, IGFBP, and RELN, were greatly upregulated in the malignant TME (Figure S5D), implicating their roles in extracellular matrix remodeling, immune modulation, and angiogenesis. Further dissection of ligand–receptor interactions identified key collagen– and laminin–integrin signaling axes as dominant communication routes between EpiC6 cells and CAFs (Figure 5C). Specifically, interactions such as COL1A1/COL1A2/COL4A1/COL4A2 with ITGA2+ITGB1, CD44, and SDC1, as well as LAMA2/LAMA4/LAMB1/LAMC1 with ITGA2+ITGB1 and CD44, were highly enriched (Figure 5C,D). These networks suggest that LAMC2^+^ EpiC6 cells may activate and sustain CAFs through matrix–integrin feedback loops, thereby reinforcing a tumor‐permissive microenvironment that supports cancer progression.

To validate these findings in situ, we performed mIHC staining on OSCC samples from betel nut chewers and nonchewers. Tumors from betel nut chewers displayed a higher abundance of LAMC2^+^/pan‐CK^+^ epithelial cells and a marked enrichment of CD74^+^ apCAFs, which were preferentially localized at the invasive front in close spatial proximity to malignant cells (Figure 5E). Furthermore, the immune microenvironment in betel nut‐associated OSCC exhibited a substantial increase in SPP1^+^/CD68^+^ macrophages compared with nonchewing samples. These macrophages were found to accumulate within CD74^+^ CAF‐rich stromal niches, forming a spatial triad surrounding LAMC2^+^ tumor cell clusters, suggesting coordinated cellular crosstalk that reinforces immune suppression and epithelial plasticity (Figure 5E).

Collectively, these results uncover a highly coordinated and spatially organized cellular communication network in betel nut‐associated OSCC. Malignant epithelial cells, CAFs, and macrophages engage in dynamic and mutually reinforcing interactions that promote tumor progression. Our findings highlight critical signaling hubs, particularly collagen– and laminin–integrin pathways, as potential therapeutic vulnerabilities, offering mechanistic insights and translational opportunities for disrupting tumor–stroma crosstalk and mitigating disease advancement.

### 2.6 Arecoline Enhances the Migratory and Invasive Potential of LAMC2^+^ OSCC Cells

**FIGURE 6:**
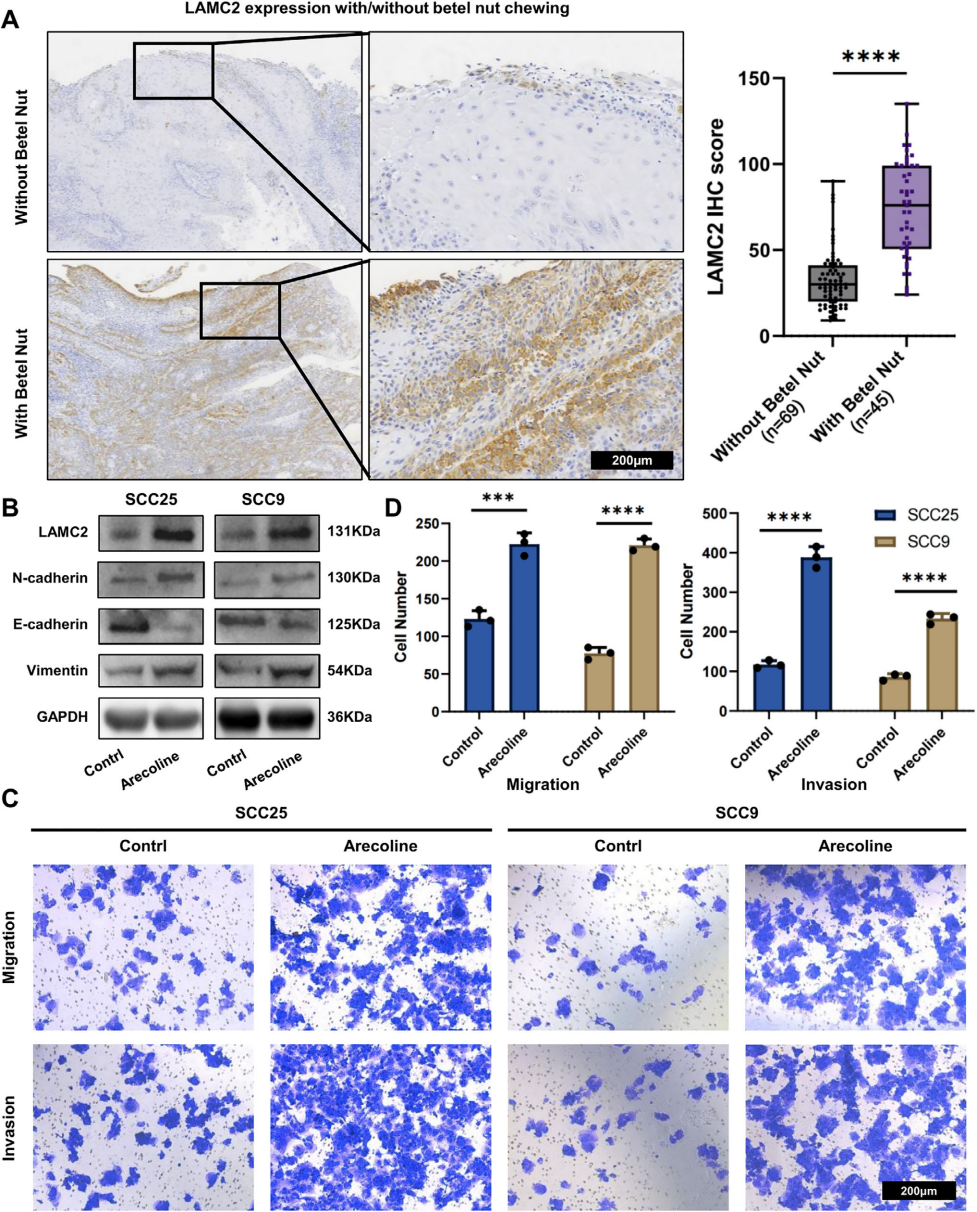
Arecoline enhances the migratory and invasive potential of LAMC2^+^ OSCC cells. (A) Representative immunohistochemical staining images showing LAMC2 expression in OSCC tissues from betel nut chewers and nonchewers. The box‐and‐whisker plot on the right summarizes LAMC2 expression levels; each dot represents an individual clinical specimen (****p < 0.0001 by Student's *t*‐test). (B) Western blot images showing the expression of LAMC2, EMT‐associated markers (N‐cadherin, E‐cadherin, and Vimentin), and GAPDH in OSCC cell lines SCC25 and SCC9 under control and arecoline‐treated conditions. (C) Representative Transwell assay images illustrating migratory and invasive capacities of SCC25 and SCC9 cells in control and arecoline‐treated groups. (D) Bar plots showing the quantification of migration and invasion assays for SCC25 and SCC9 cells under control and arecoline‐treated conditions (*n* = 3, ***p < 0.001, ****p < 0.0001 by Student's *t*‐test).

To further elucidate the association between the phenotypic characteristics of LAMC2^+^ OSCC cells and betel nut chewing, we analyzed a cohort of 114 OSCC clinical specimens (Table S12), which were stratified into betel nut chewers (*n* = 45) and nonchewers (*n* = 69). Immunohistochemical analysis revealed that LAMC2 expression was significantly elevated in tumor tissues derived from betel nut chewers compared with those from nonchewers (Figure 6A). These findings suggest a strong clinical correlation between betel nut exposure and LAMC2 upregulation in OSCC, implicating LAMC2 as a potential mediator of betel nut‐associated tumor aggressiveness.

To validate these observations in vitro, we employed the OSCC cell lines SCC9 and SCC25 and treated them with arecoline to mimic the pathological effects of betel nut chewing. Western blot analysis demonstrated that arecoline treatment markedly increased LAMC2 protein levels relative to untreated controls. Concomitantly, the epithelial marker E‐cadherin was substantially downregulated, whereas the mesenchymal markers N‐cadherin and Vimentin were robustly upregulated (Figure 6B), indicating that arecoline robustly promotes EMT in OSCC cells. Given the established role of EMT in promoting tumor invasion and metastasis, we next assessed the functional consequences of arecoline treatment using Transwell migration and invasion assays. As expected, arecoline‐treated SCC9 and SCC25 cells exhibited a pronounced increase in migratory and invasive capacities compared with control cells (Figure 6C,D), supporting the notion that arecoline enhances the metastatic potential of LAMC2^+^ OSCC cells.

Collectively, these results demonstrate that arecoline exposure significantly upregulates LAMC2 expression in OSCC cells and promotes EMT, thereby enhancing their migratory and invasive abilities. These findings provide mechanistic insight into how betel nut chewing contributes to OSCC progression and identify LAMC2 as a critical molecular link between environmental carcinogen exposure and aggressive tumor behavior.

## 3 Discussion

Betel nut chewing is a widespread addictive habit, affecting over 600 million individuals globally and representing a vital etiological factor for OSCC [20, 21]. Here, we present a comprehensive single‐cell and spatial transcriptomic dissection of the TME in betel nut‐associated OSCC, uncovering distinct cellular ecosystems, spatial niches, and regulatory programs that drive malignant transformation and immune modulation. These findings expand our understanding of how habitual betel nut exposure reshapes the oral mucosal landscape to facilitate OSCC progression. By integrating single‐cell and spatial transcriptomic data, our study reveals profound epithelial heterogeneity and lineage dynamics underlying betel nut‐associated OSCC. We identified a malignant epithelial subpopulation, LAMC2^+^ EpiC6 cells, markedly enriched in betel nut users and characterized by strong activation of EMT, extracellular matrix remodeling, invasion, and metastatic programs. The increased abundance and terminal positioning of EpiC6 cells in the pseudotime trajectory suggest that these cells represent a terminally differentiated, highly invasive state in the epithelial lineage, potentially arising under chronic betel nut exposure. LAMC2^+^ EpiC6 cells exhibited elevated activity of EMT‐associated TFs such as SNAI1, RUNX2, SMAD4, and PRRX1, consistent with previous findings that implicate these regulators in EMT‐mediated tumor progression [22]. The increased abundance of highly proliferative EpiC7 cells, expressing cell cycle‐related genes such as *TOP2A* and *MCM5*, further underscores the proliferative stimulus driven by carcinogenic components of betel nut, such as arecoline [14]. These results align with previous mouse model studies demonstrating MYC pathway activation in epithelial stem and keratinocyte compartments during carcinogen‐induced oral tumorigenesis [14].

The TME in betel nut‐associated OSCC is characterized by profound stromal reprogramming and immune modulation. Our data reveal marked expansion of CAFs, particularly apCAFs, along with a reduction in cytotoxic T/NK cells and increased infiltration of metabolically reprogrammed SPP1^+^ macrophages. A prominent feature of the TME in betel nut‐associated OSCC is the expansion and functional polarization of CAF subtypes, particularly apCAFs. These subpopulations exhibit enriched expression of immunomodulatory and ECM‐remodeling genes, suggesting their dual roles in immune suppression and tumor invasion. Our findings are consistent with prior reports of pEMT programs serving as intermediates in tumor–stroma transitions, as well as CAF1 and CAF2 phenotypes exhibiting spatially distinct, functional roles in ECM remodeling and immune regulation [23, 24, 25]. The CAF2 subset (apCAFs/iCAFs), in particular, demonstrated strong immune‐related transcriptional programs and enriched ligand–receptor interactions with B cells, macrophages, and DCs, potentially contributing to tertiary lymphoid structure (TLS) formation. This mirrors the immune‐rich microenvironment reported in OSF‐derived OSCC and supports the hypothesis that chronic inflammation fosters an immune‐reactive TME with potential prognostic implications [26, 27, 28]. Additionally, the observed enrichment of lymphatic endothelial cells within tumor tissues underscores their potential contribution to metastasis and lymphatic remodeling in the setting of chronic betel nut exposure.

In particular, SPP1^+^ C18 macrophages exhibited a highly immunosuppressive, proangiogenic, and metabolically active phenotype, marked by upregulation of *MMP9*, *VEGFB*, and glycolytic genes, consistent with an M2‐like polarization state known to suppress anti‐tumor immunity [11]. These macrophages accumulated predominantly within tumor tissues of betel nut users, indicating their role in establishing a metabolically adaptive and immune‐evasive microenvironment. Our identification of expanded lymphatic endothelial cells expressing *FLT4*, *PROX1*, and *VEGFC* further suggests a mechanism for enhanced lymphangiogenesis and metastatic spread in betel nut‐associated tumors. This aligns with previous findings indicating that endothelial and CAF subtypes contribute to ECM remodeling, angiogenesis, and immune cell exclusion [11, 29, 30]. Notably, the iCAF subset in our data expressed *CXCL12*, *CCL19*, and *PDGFRA*, consistent with their known role in recruiting regulatory T cells and suppressing CD8^+^ T cell infiltration through CXCL12/CXCR4 signaling [11, 29, 30]. Together, these observations reinforce the notion that betel nut exposure induces a fibrotic, immune‐excluded, and proangiogenic microenvironment that fosters immune escape and aggressive tumor progression.

Our cell–cell communication analysis revealed that LAMC2^+^ EpiC6 tumor cells establish extensive crosstalk with CAFs via ligand–receptor pairs such as COL1A1–ITGA2, COL1A1–CD44, and laminin‐mediated interactions. This aligns with previous findings in OSF‐derived OSCC, where tumor cells exhibited a phenotypic transition from proliferative epithelial states to mesenchymal‐like CAF‐like identities [31, 32]. These signaling axes likely reinforce CAF activation and establish a feedforward loop sustaining tumor‐promoting inflammation and fibrosis. Previous studies have shown that CAFs are major sources of TGF‐β1, which in turn activates EMT and promotes tumor invasion through the TGF‐β/Smad signaling axis [10]. The enrichment of myCAFs, expressing *COL1A1*, *ACTA2*, and *TAGLN*, around tumor nests, and iCAFs in peripheral fibrotic regions, supports a spatially coordinated role of CAFs in enhancing ECM stiffness, immune suppression, and tumor dissemination [10, 22]. Moreover, pseudotime analysis of our data supports the potential transition of epithelial cells toward a mesenchymal phenotype (partial EMT), consistent with prior findings that epithelial–CAF interactions, matrix stiffness, and metabolic alterations can drive epithelial plasticity and promote malignant conversion [22, 33]. The combined evidence suggests that reciprocal signaling between LAMC2^+^ EpiC6 cells and CAF subsets orchestrates a tumor‐promoting loop involving ECM remodeling, immune evasion, and EMT. Targeting key mediators such as LAMC2, COL1A1, or CAF‐related transcriptional regulators (SMAD4, RUNX2, TCF21) may offer therapeutic opportunities to disrupt this malignant axis in betel nut‐associated OSCC.

Despite the insights provided by this integrative single‐cell and spatial transcriptomic analysis, several limitations should be acknowledged. First, the relatively small cohort size, dictated by the limited availability of well‐annotated betel nut‐associated OSCC specimens suitable for formalin‐fixed, paraffin‐embedded (FFPE)‐based multiomics profiling, may constrain the generalizability of our findings. Although consistent transcriptional and spatial patterns were observed across samples, validation in larger, independent multicenter cohorts will be essential to confirm the robustness of the identified cellular states and tumor microenvironmental interactions. Second, the use of FFPE‐derived tissues, while enabling the inclusion of clinically valuable archived specimens, is inherently associated with partial RNA degradation and reduced transcript capture efficiency compared with fresh‐frozen samples, future studies incorporating fresh‐frozen tissues may further improve transcriptomic resolution and enable more comprehensive detection of low‐abundance transcripts. Third, while our analyses reveal several keys signaling pathways (e.g., SPP1 or COL1A1–CD44 axes), the present study primarily provides correlative evidence. Although we have supplemented our findings with targeted in vitro validation, additional functional studies using genetic or pharmacological perturbation in in vitro and in vivo models will be necessary to establish definitive causal relationships underlying these cell–cell communication networks. Finally, our study focuses specifically on betel nut‐associated OSCC, an etiologically distinct but understudied subtype. Comparative analyses incorporating tobacco‐ or alcohol‐associated OSCC, as well as longitudinal sampling to capture betel nut‐induced molecular evolution during tumor progression, would further elucidate etiology‐specific mechanisms of TME remodeling and disease progression.

In conclusion, our study reveals a distinct immunosuppressive and stromal‐reinforced TME in betel nut‐associated OSCC, shaped by CAF expansion, metabolically adapted macrophages, dysregulated B cell immunity, and epithelial–stromal communication. This Integrating multiomics approaches with functional studies may uncover novel biomarkers and therapeutic targets, addressing the unmet clinical need for effective interventions in this high‐risk population.

## 4 Materials and Methods

### 4.1 Clinical Sample Collection and Processing

Primary tumor and matched adjacent nontumorous tissues were obtained from six OSCC patients, including three individuals with a history of betel nut chewing and three without. Clinical details are provided in Table S1. Primary tumor specimens and tumor‐adjacent tissues from OSCC patients were collected immediately following surgical resection and fixed in 10% neutral‐buffered formalin at room temperature for 24–48 h. Fixed tissues were then dehydrated through a graded ethanol series, cleared in xylene, and embedded in paraffin using standard histopathological procedures. Paraffin blocks were stored at room temperature until sectioning for downstream applications.

### 4.2 Tissue Dissociation and Generation of Single‐cell Suspensions

FFPE tissue sections were processed following the 10× Genomics demonstrated protocol (CG000784, Rev B). Briefly, 25–50 µm tissue scrolls were sectioned from FFPE blocks, transferred into 1.5 mL microcentrifuge tubes, and subjected to a sequential deparaffinization and rehydration process using xylene and graded ethanol solutions. After rehydration in PBS on ice, nuclei isolation was performed using a pestle‐based mechanical dissociation method. Tissues were enzymatically digested in a Dissociation Enzyme Mix containing Liberase TH in RPMI medium, incubated at 37°C for 45 min with intermittent mixing. The resulting suspension was passed through a 30 µm cell strainer, followed by centrifugation at 850 rcf for 5 min at 4°C. The nuclear pellet was resuspended in chilled Quenching Buffer B (10× Genomics), and nuclei concentration was determined using a fluorescent nucleic acid stain (e.g., Propidium Iodide) and a Cellaca MX or equivalent automated cell counter. Only samples meeting the minimum input threshold of 25,000 nuclei were used for downstream GEM‐X Flex Gene Expression profiling, with an optimal target of 300,000 nuclei per hybridization reaction.

### 4.3 Sing‐Cell RNA‐seq Library Preparation and Sequencing

Single‐cell transcriptomic libraries were prepared using the 10× Genomics Chromium Fixed RNA Profiling platform according to the manufacturer's protocol. Briefly, cell or nuclei suspensions were fixed using 4% paraformaldehyde to preserve RNA integrity and cellular morphology. Target mRNA molecules were captured using a probe hybridization strategy employing two complementary 25‐nucleotide probes designed to hybridize adjacently on each target transcript. One probe contains a partial capture sequence for binding to oligonucleotides on gel beads, and the other includes a partial sequencing adapter to facilitate library construction. Following hybridization and washing, the cell suspensions were diluted to appropriate concentrations and loaded into the chromium controller to generate gel beads‐in‐emulsion (GEMs). Successful GEM formation was visually verified, and GEMs were transferred to PCR tubes for reverse transcription and cDNA amplification. The resulting libraries were subjected to quality control prior to sequencing. Sequencing was performed on an Illumina HiSeq or NovaSeq platform using paired‐end 150 bp reads (PE150). A minimum sequencing depth of 15,000 read pairs per cell was targeted, with each sample expected to yield transcriptomic profiles for up to 8000–10,000 individual cells. Increased sequencing depth was employed when needed to improve gene detection sensitivity.

### 4.4 Sing‐Cell RNA‐seq Data Preprocessing

Raw sequencing data generated using the 10× Genomics Chromium platform were processed with the Cell Ranger pipeline (v7.0.0; 10× Genomics) to perform demultiplexing, barcode and unique molecular identifiers (UMIs) extraction, read alignment, and gene quantification. Specifically, FASTQ files were generated from the raw base call files using the “cellranger mkfastq” module, followed by alignment of reads to the GRCh38 human reference genome using the STAR [34] aligner integrated within the “cellranger count” function. The resulting gene expression matrices were subsequently imported into the Seurat [35] R package (v4.4.0) for quality control and downstream analysis. Cells were filtered based on standard quality control metrics to exclude potential doublets and low‐quality cells. Specifically, cells with fewer than 200 detected genes or with >30% of total UMI counts derived from mitochondrial genes were removed. All quality control and preprocessing steps were performed using default parameters unless otherwise specified.

### 4.5 Batch Effect Correction and Multiple Dataset Integration

To enable direct comparison of cellular identities and proportions across different biological conditions, we performed multiple dataset integration and batch effect correction using the Seurat R package (v4.4.0), following the anchor‐based integration framework described previously [35]. This approach enables the alignment of shared cell states across datasets while preserving biological heterogeneity. Briefly, each dataset was independently preprocessed using the “SCTransform” function, which performs variance‐stabilizing normalization and scaling based on regularized negative binomial regression. To mitigate the influence of mitochondrial gene expression on downstream analyses, we regressed out the proportion of mitochondrial transcripts by setting the parameter “vars.to.regress = ‘percent.mito’.” Highly variable features were then identified across datasets using the “SelectIntegrationFeatures” function, with the number of features set to 3000 to capture the most informative gene expression signals. This was followed by data preparation for integration via “PrepSCTIntegration” function, which computes the necessary residuals and normalization parameters for each dataset. Integration anchors, which represent correspondences between biologically similar cells across datasets, were identified using the “FindIntegrationAnchors” function. We specified “normalization.method = ‘SCT’” to ensure consistency with SCTransform‐normalized inputs and used CCA as the dimensionality reduction method by setting “reduction = ‘cca’.” Finally, the integrated expression matrix was constructed using the “IntegrateData” function, which uses the previously defined anchors to correct for batch‐specific technical variation. The resulting integrated object served as the foundation for unified downstream analyses, including dimensionality reduction, clustering, and differential expression testing.

### 4.6 Dimensionality Reduction and Unsupervised Clustering of scRNA‐seq Data

To reduce the dimensionality of the datasets, the “RunPCA” function was conducted with default parameters on the SCTransform‐normalized and variance‐stabilized expression matrix. Next, the “ElbowPlot” function was used to identify the true dimensionality of each dataset, as recommended by the Seurat developers, and the top 30 principal components (PCs) were selected for subsequent analyses. Cell clustering was performed by constructing a shared nearest neighbor (SNN) graph using the “FindNeighbors” function, followed by modularity optimization‐based community detection with the “FindClusters” function. A resolution parameter of 1.2 was used to balance granularity and biological interpretability of cell clusters. For visualization of the global cellular landscape, we applied nonlinear dimensional reduction using UMAP through the “RunUMAP” function with default settings. This allowed projection of high‐dimensional transcriptomic data into two‐dimensional space, facilitating intuitive exploration of cellular heterogeneity.

### 4.7 Cell Type Annotation and Cluster Marker Identification

To characterize the molecular identity of each cell cluster, we performed differential gene expression analysis using Seurat's “FindAllMarkers” function. We specified “logfc.threshold = 0.5, min.pct = 0.25, and *p*_val_adj < 0.05” to identify robust cluster‐enriched marker genes. Differential expression testing was conducted using the Wilcoxon rank‐sum test, comparing each cluster against all others.

Clusters were annotated manually by comparing the expression patterns of canonical lineage markers associated with known immune, epithelial, and stromal cell types. Cell type labels were assigned based on the presence, specificity, and relative abundance of these markers. Clusters coexpressing markers from multiple distinct lineages were flagged as potential doublets or multiplets and were excluded from downstream analysis. Similarly, clusters with low transcriptomic complexity or lacking expression of recognizable canonical markers were classified as low‐quality or unclassifiable and were also removed from subsequent interpretation. These filtering steps ensured that downstream analyses focused on biologically meaningful and high‐confidence cell populations.

### 4.8 GO Functional Enrichment Analysis

To gain insight into the biological functions and pathways associated with each cell cluster, we performed GO enrichment analyses using the clusterProfiler [36] (v4.10.1) R package to identify biological pathways that were enriched in a certain gene list more than that would be expected by chance. GO terms with *p* value < 0.05 were considered significantly enriched. To facilitate interpretation of the enrichment results, we used the “cnetplot” and “emapplot” functions from the clusterProfiler package to visualize gene‐term relationships and pathway connectivity. Additionally, the “ClusterGVis” R package (https://github.com/junjunlab/ClusterGVis, v0.1.2) was employed for interactive and customizable visualization of enrichment results, allowing clearer interpretation of functional associations among gene clusters.

### 4.9 UCell Gene Signature Enrichment Analysis

To assess the functional states of distinct cell subclusters at single‐cell resolution, we performed gene signature enrichment analysis using the UCell [37] (v2.6.2) R package, which computes robust, rank‐based enrichment scores for predefined gene sets across individual cells. Tumor‐associated gene signatures were retrieved from the CancerSEA (http://biocc.hrbmu.edu.cn/CancerSEA/) database (Table S5), while macrophage‐ and metabolism‐related gene signatures (Table S9) were curated from published literature [38]. The “AddModuleScore_UCell” function was applied to epithelial and macrophage subpopulations to quantify gene set activity scores, enabling comparative evaluation of functional characteristics such as proliferation, hypoxia, immune response, and metabolic reprogramming among distinct subclusters.

### 4.10 TF Activity and Coexpression Network Analysis

TF regulatory activity was inferred using the decoupleR [39] package (v2.8.0), which integrates gene expression with known TF–target relationships to estimate the activity of individual TFs in each cell. The resulting TF activity matrix was visualized using the “pheatmap” R package (v1.0.12), highlighting differences in regulatory landscapes among epithelial and CAF subclusters. To further investigate regulatory programs in the LAMC2^+^ epithelial subpopulation (EpiC6), TF coexpression analysis was performed using the GENIE3 [40] algorithm (v1.24.0), which applies tree‐based ensemble methods to infer gene regulatory networks. Network visualization was conducted using the “ggraph” package (v2.2.1), illustrating key TF‐gene interactions. For CAF subclusters, TF‐specific protein–protein interaction networks were constructed using the STRING (https://string‐db.org) database, which aggregates experimental and predicted interactions. The resulting regulatory networks were visualized using Cytoscape [41] (v3.10.3) with a hierarchical layout to reveal hierarchical and functional relationships among cluster‐specific TFs.

### 4.11 Pseudotime Trajectory Inference

To reconstruct potential lineage relationships and infer dynamic transcriptional changes underlying epithelial and macrophage cell state transitions, pseudotime trajectory analysis was performed using Monocle2 [16, 17] (v2.30.1). Cluster‐specific expression matrices obtained from Seurat were used to construct “CellDataSet” objects via the “newCellDataSet” function, with raw counts from the Seurat RNA assay as input. Lowly expressed genes were filtered using the “detectGenes” function (min_expr = 0.1). Dimensionality reduction was conducted using the “DDRTree” algorithm via the “reduceDimension” function (reduction_method = “DDRTree”), followed by cell ordering using “orderCells” function. Trajectories were visualized with “plot_cell_trajectory,” and gene expression dynamics across pseudotime were examined using “plot_pseudotime_heatmap,” which clustered genes into coexpression modules. Branch‐specific transcriptional programs were further characterized using Branched Expression Analysis Modeling, enabling differential expression analysis across lineage branches. Functional enrichment and trajectory‐based heatmaps were visualized using the “ClusterGVis” R package, providing insights into key regulators and biological processes governing state transitions.

### 4.12 Cell–Cell Communication Analysis

To investigate intercellular signaling and infer the functional crosstalk among different cell populations, we applied CellChat [19] (v2.1.2), a comprehensive framework that decodes cell–cell communication networks from single‐cell transcriptomic data. CellChat infers ligand–receptor interactions by integrating prior knowledge of signaling pathways with cell‐type‐specific gene expression profiles. The resulting signaling networks allowed us to identify key signaling axes, infer directional interactions between sender and receiver cell types, and assess the contribution of each cell population to specific signaling pathways. This analysis provided mechanistic insights into the coordination of tumor and stromal cell functions within the OSCC TME.

### 4.13 Spatial Transcriptomics Library Preparation and Sequencing

Two pairs of samples, each comprising tumor and matched adjacent nontumor tissue, were selected for spatial transcriptomic analysis (Table S1). Tissue size, morphology, and preservation quality of the samples were carefully evaluated to ensure optimal placement within the 6.5 × 6.5 mm^2^ capture area of the Visium Spatial Gene Expression Slides (10× Genomics). Only FFPE tissue blocks with adequate RNA quality, defined by DV200 values >30%, were included for downstream analysis. Spatial transcriptomic profiling was performed on four FFPE samples using the Visium Spatial Gene Expression for FFPE workflow coupled with the CytAssist platform (10× Genomics). FFPE sections (5 µm thickness) were processed according to the manufacturer's Tissue Preparation Guide (CG000518), including deparaffinization, antigen retrieval, and heat‐induced decrosslinking at 65°C for 1 h using the proprietary decrosslinking buffer supplied by 10× Genomics. This step was designed to reverse formaldehyde‐induced crosslinks while preserving fragmented RNA suitable for probe‐based capture. Transcript detection was achieved using the 10× Genomics Fixed RNA Profiling whole‐transcriptome probe panel, which targets approximately 18,000–20,000 human protein‐coding genes through a hybridization‐based strategy optimized for FFPE‐derived RNA fragments. Probe hybridization, ligation, and barcoding were performed following the Visium CytAssist Spatial Gene Expression Reagent Kit protocol. Each capture area contained approximately 5000 spatially barcoded spots, each with a diameter of 55 µm and a center‐to‐center distance of 100 µm. Brightfield imaging and hematoxylin and eosin staining were conducted according to the Spatial Applications Imaging Guidelines (CG000521) and staining instructions (CG000520), respectively. Library construction and sequencing were performed following the manufacturer's protocol (CG000495). Library quality and capture performance were evaluated using standard 10× Genomics quality control metrics, including the number of detected genes and UMIs per spot, as well as the fraction of reads mapped to targeted probes. For each FFPE sample, FASTQ files and manually aligned histological images were processed using Space Ranger (v2.0.0; 10× Genomics) with the prebuilt GRCh38 reference genome (refdata‐cellranger‐GRCh38‐3.0.0) to generate spatially resolved gene expression matrices.

### 4.14 Spatial Transcriptomics Data Processing

All spatial transcriptomic data were processed and analyzed using the Seurat (v4.4.0) R package unless otherwise stated. To ensure the inclusion of high‐quality and biologically informative features, we first applied stringent quality control criteria to exclude spatial spots with fewer than 100 detected genes (nFeature < 100) or with mitochondrial gene content exceeding 20% (percent_mito > 20), thereby reducing the influence of low‐abundance or noninformative transcripts. To mitigate technical variation across slides, each sample was individually normalized and variance‐stabilized using the “SCTransform” algorithm, which employs regularized negative binomial regression to correct for sequencing depth and technical noise. Following normalization, datasets from the four spatial transcriptomic samples were integrated using the harmony [42] (v1.2.0) R package to correct for inter‐sample batch effects. Specifically, 3000 highly variable features were identified across samples and used for integration. The “RunHarmony” function was executed with the parameters group.by.vars = “Sample” and assay.use = “SCT” to generate a harmonized expression matrix that facilitates joint downstream analyses while preserving sample‐specific biological information. Dimensionality reduction was performed on the integrated dataset by PCA, and the top 30 PCs were retained based on variance explained and elbow plot inspection.

Spatial clusters were identified by constructing a SNN graph using the “FindNeighbors” function, followed by modularity‐based clustering via “FindClusters,” with a resolution parameter set to 0.5 to strike a balance between granularity and interpretability of spatial domains. Low‐dimensional embedding and visualization of the spatial architecture were achieved using UMAP, and cluster assignments were visualized in both nonspatial and spatial contexts using Seurat's “DimPlot” and “SpatialDimPlot” functions, respectively. To investigate gene expression patterns in situ, spatially resolved expression maps were generated using the “SpatialFeaturePlot” function, enabling the direct visualization of transcript abundance within histological architecture. To annotate spatial spots with putative cell type identities, we leveraged matched single‐cell RNA‐seq datasets through a label transfer strategy. First, integration anchors between spatial transcriptomic data and scRNA‐seq reference were identified using the “FindTransferAnchors” function with normalization.method = “SCT.” Subsequently, cell type labels from the annotated scRNA‐seq dataset were projected onto spatial spots using the “TransferData” function, resulting in spatially resolved maps of cell type distributions. This integrative approach enabled high‐resolution annotation of tissue regions with biologically meaningful cell‐type information, enhancing the interpretability of spatial transcriptomic data.

### 4.15 mIHC Staining

FFPE sections of OSCC tissue were stained using a mIHC kit (Akoya; Cat# NEL811001KT), following the manufacturer's instructions. Endogenous peroxidase activity was quenched with 3% hydrogen peroxide for 10 min. Heat‐induced epitope retrieval was performed, followed by blocking with 5% bovine serum albumin before each primary antibody incubation. The primary antibodies used were: LAMC2 (1:300, ab210959; Abcam), CD74 (1:200, ABB6704; Huilanbio), Pan‐CK (1:5000, 26411‐1‐AP; Proteintech), SPP1 (1:300, 83341‐2‐RR; Proteintech), and CD68 (1:1000, ab955; Abcam), each targeting specific antigens. Detection was performed using HRP‐conjugated secondary reagents with fluorophores at 480, 520, 570, 620, and 690 nm. Cell nuclei were counterstained with DAPI (Solarbio; Cat# C0065‐50 mL).

### 4.16 OSCC Clinical Cohort Collection

A cohort comprising 114 OSCC tissue samples was collected from the Department of Oral and Maxillofacial Surgery, The First Affiliated Hospital of Sun Yat‐sen University, between January 2022 and December 2024. Pathological diagnosis and clinicopathological parameters were determined according to the eighth edition of the American Joint Committee on Cancer staging system. Clinicopathological characteristics of the 114 patients, including sex, age, smoking history, alcohol consumption, betel nut chewing history, tumor site, histological differentiation, and clinical TNM stage, were collected for analysis. This study was approved by the Ethics Committee of The First Affiliated Hospital of Sun Yat‐sen University (Approval No. [2024]821) and was conducted in accordance with the Declaration of Helsinki.

### 4.17 IHC Staining

IHC was performed following antigen retrieval and blocking of endogenous peroxidase activity with 3% H_2_O_2_. Tissue sections were incubated overnight at 4°C with a primary antibody against LAMC2 (1:500, ab210959; Abcam). After washing, sections were incubated with the appropriate secondary antibody followed by a streptavidin–peroxidase complex (SA1022; Boster, Wuhan, China). Immunoreactivity was visualized using 3,3′‐diaminobenzidine (DAB; Boster) and counterstained with hematoxylin. IHC scores were calculated by multiplying the staining intensity score by the percentage of positively stained cells, yielding a final score ranging from 0 to 300.

### 4.18 Cell Culture and Arecoline Treatment

SCC9 and SCC25 cells were obtained from the American Type Culture Collection. Cells were cultured in DMEM/F12 (Gibco) supplemented with 10% fetal bovine serum and 1% penicillin–streptomycin, and maintained at 37°C in a humidified atmosphere containing 5% CO_2_. Routine mycoplasma testing was performed using the PlasmoTest mycoplasma detection kit (InvivoGen). SCC9 and SCC25 cells were treated with arecoline. When cell confluence reached approximately 80%, cells were exposed to arecoline at approximately half of the IC_50_ concentration (80 µg/mL). The culture medium was replaced every 12 h while maintaining this concentration, and cells were continuously stimulated until confluence again reached about 80%, followed by passaging. Subsequently, arecoline was added to the culture medium with stepwise increases of 20 µg/mL at each stage, ultimately reaching a final concentration of 160 µg/mL for sustained stimulation of OSCC cells, thereby generating arecoline‐induced model cells for subsequent experiments. In this set of experiments, cells were divided into two groups: the experimental group consisted of arecoline‐induced model cells (arecoline group), while the control group was treated with an equal volume of double‐distilled water (control group).

### 4.19 Western Blot Analysis

Cells were lysed in radioimmunoprecipitation assay (RIPA) buffer (Beyotime) supplemented with a protease inhibitor cocktail and, when indicated, phosphatase inhibitors (Beyotime). Equal amounts of protein were separated by 10% sodium dodecyl sulfate–polyacrylamide gel electrophoresis and transferred onto polyvinylidene fluoride membranes. After blocking with 5% skimmed milk, membranes were incubated with primary antibodies against LAMC2 (1:500, ab210959; Abcam), Vimentin (1:5000, 10366‐1‐AP; Proteintech), E‐cadherin (1:5000, 20874‐1‐AP; Proteintech), N‐cadherin (1:8000, 22018‐1‐AP; Proteintech), and GAPDH (1:20,000, 10494‐1‐AP; Proteintech). Membranes were then incubated with HRP‐conjugated anti‐rabbit (1:5000, SA00001‐2) or anti‐mouse (1:5000, SA00001‐1) secondary antibodies (Proteintech). Protein signals were visualized using the Tanon 5200 Multi Intelligent Imaging System (Tanon, Shanghai, China).

### 4.20 Transwell Cell Migration and Invasion Assays

For migration assays, 5 × 10^4^ cells suspended in 200 µL of serum‐free DMEM/F12 were seeded into the upper chambers of Transwell inserts (Corning, China), while the lower chambers were filled with 500 µL of DMEM/F12 containing 1% FBS. For invasion assays, the Transwell inserts were precoated with 5% Matrigel (BioCoat, China), and cells were seeded under the same conditions as described for migration assays. After 48 or 72 h of incubation, nonmigrated or noninvaded cells in the upper chambers were removed. Cells that had migrated or invaded to the lower surface were fixed, stained with crystal violet, and quantified under a microscope by counting five random fields.

### 4.21 Statistical Analysis

All statistical analyses were performed using R version 4.3.3 and GraphPad Prism 9 (GraphPad Software, Inc., CA, USA). Student's *t*‐test, Wilcoxon rank‐sum test, and Kruskal–Wallis test was utilized in this study. A two‐tailed *p* value < 0.05 was considered statistically significant unless otherwise noted (ns, *p* ≥ 0.05; *, *p* < 0.05; **, *p* < 0.01; ***, *p* < 0.001; ****, *p* < 0.0001).

## Supporting Information

Additional supporting information can be found online in the Supporting Information section.

## Author Contributions

Conceptualization: Wei Dong, Shuojin Huang, Yijun Wu, Congyuan Cao, Jiaxue Li, Qianting He, and Anxun Wang. Methodology: Wei Dong, Shuojin Huang, Yijun Wu, Congyuan Cao, and Jiaxue Li. Data analysis and curation: Wei Dong, Shuojin Huang, Yijun Wu, Congyuan Cao, and Jiaxue Li. Investigation and validation: Shuojin Huang and Qianting He. Resources: Qianting He and Anxun Wang. Writing – original draft: Wei Dong, Shuojin Huang, Qianting He, and Anxun Wang. Writing – review and editing: Qianting He and Anxun Wang. Supervision and funding acquisition: Anxun Wang and Qianting He. All authors read and approved of the final manuscript.

## Conflicts of Interest

The authors declare no conflicts of interest.

## Ethics Statement

The ethical, medical, and scientific aspects of the research were reviewed and approved by the Ethics Committee of the First Affiliated Hospital, Sun Yat‐Sen University before initiation (ethical approval number: [2024]821).

## Supporting information



Figure S1. Comprehensive single‐cell and spatial transcriptomic profiling of the TME in betel nut‐associated OSCC, related to Figure 1.
**(A–D)** UMAP plots visualizing single‐cell transcriptomic data, colored by individual samples (**A**), sample group (**B**), sample type (**C**), and major cell types (**D**) in betel nut‐associated OSCC. Each dot represents an individual cell, and colors denote the individual samples, sample groups, sample type, and major cell types.
**(E)** Bubble plots showing the relative average expression of canonical marker genes (*x*‐axis) across the cell clusters (*y*‐axis). Dot size indicates the proportion of cells expressing the gene, while color reflects the normalized expression level.
**(F)** Stacked bar plots illustrating the distribution of major cell types between non‐betel nut and betel nut groups. Colors represent different cell types.
**(G, H)** UMAP visualization of spatial transcriptomic spots from different samples (**G**) and cell clusters (**H**) in betel nut‐associated OSCC. Colors represent different samples and cell clusters.
**(I)** Spatial plots depicting the distribution of cell clusters across different samples. Colors represent different cell clusters.
**Figure S2**. Characterization of a highly invasive LAMC2^+^ malignant epithelial subpopulation in betel nut‐associated OSCC, related to Figure 2.
(**A**) Bubble plots showing the relative average expression of top 5 marker genes (*x*‐axis) across the epithelial cell subclusters (*y*‐axis). Dot size indicates the proportion of cells expressing the gene, while color reflects the normalized expression level.
(**B**) Stacked bar plots quantifying the relative abundance of 8 epithelial subclusters between non‐betel nut and betel nut groups. Colors represent different cell clusters.
(**C**) Clustered heatmap of UCell signature scores across epithelial subclusters. Each row represents a tumor‐associated molecular signature (e.g., EMT, invasion, hypoxia), and each column represents an epithelial subcluster.
**(D)** Pseudotime trajectory plot visualizing the inferred differentiation landscape of epithelial cells, colored by cell group types.
**(E)** Ridge plot showing the inferred epithelial cell differentiation trajectory among different cell subclusters. Colors represent different cell subclusters.
**(F)** Spatial transcriptomic plots illustrating the expression and coexpression patterns of *LAMC2* and *KRT5* genes in different samples, with LAMC2^+^ epithelial cells marked by irregular outlines.
**(G)** Violin plot showing the expression levels of *LAMC2* in different samples. Each dot represents an individual cell, and colors represent different samples.
**(H)** Box plot comparing the abundance of LAMC2*^+^* epithelial cells between betel nut chewers and nonchewers (n = 5, ****p < 0.0001 by Student's *t*‐test).
**Figure S3**. Functional heterogeneity of endothelial cells in the TME of betel nut‐associated oral cancer, related to Figure 3.
(**A**) UMAP plot showing the three transcriptionally distinct endothelial cell subclusters. Colors represent different cell clusters.
**(B)** Violin plots comparing expression levels of key vascular endothelial and lymphatic endothelial markers across the three subclusters. Each dot represents an individual cell, and colors represent different cell subclusters.
**(C)** Stacked bar plots showing the proportional representation of endothelial subclusters across four different sample group types. Colors represent different cell subclusters.
**(D)** Heatmap of cell cluster‐specific marker gene expression profiles with corresponding GO term enrichment (right).
**(E)** Heatmap depicting the expression of key genes associated with endothelial cell‐associated functional programs across three endothelial subclusters.
**Figure S4**. Profiling immune cell heterogeneity and functional diversity in the TME of betel nut‐associated OSCC, related to Figure 4.
**(A)** UMAP plot showing the three transcriptionally distinct DC cell subclusters in betel nut‐associated OSCC. Colors represent different cell clusters.
**(B)** Bubble plots showing the relative average expression of canonical marker genes (*x*‐axis) across the DC cell subclusters (*y*‐axis). Dot size indicates the proportion of cells expressing the gene, while color reflects the normalized expression level.
**(C)** Stacked bar plot displaying the distribution of DC subclusters across four different sample group types. Colors represent different cell subclusters.
**(D)** Heatmap of DC cell cluster‐specific marker gene expression profiles, annotated with enriched GO terms (right).
**(E)** UMAP plot showing the five B/plasma cell subclusters in betel nut‐associated OSCC. Colors represent different cell clusters.
**(F)** Heatmap displaying the average expression profiles of marker genes across five B/plasma cell subclusters.
**(G)** Stacked bar plot showing proportion of B/plasma cell subtypes across four different sample group types. Colors represent different cell subclusters.
**(H)** Functional enrichment network of GO terms and associated genes across different B/plasma cell subclusters. Colors represent different cell clusters.
**Figure S5**. Malignant epithelium–CAF crosstalk drives tumor progression in betel nut‐associated OSCC, related to Figure 5.
**(A, B)** Bar plots quantifying the total number of inferred ligand–receptor interactions (**A**) and the overall interaction strength (**B**) among TME cell types in betel nut‐associated OSCC.
**(C)** Circular interaction network comparing differential intercellular communication between betel nut‐associated and non‐betel nut‐associated OSCC among epithelial cells, endothelial cells, CAFs, and macrophages. Node colors represent cell subclusters, and edge colors denote differential interaction strength, with red representing increased interaction strength in betel nut tumor samples, blue represents decreased interaction strength in betel nut tumor samples.
**(D)** Heatmap summarizing the strength of overall signaling pathways among different cell clusters in four different sample group types. Each row corresponds to a specific signaling pathway, and columns represent recipient clusters. Cell type identities are annotated below.

Supplementary Table S1. Detailed baseline information of patients included in scRNA sequencing and spatial transcriptomic.
**Supplementary Table S2**. Differential expression genes (DEGs) of nine major cell types in scRNA‐seq data.
**Supplementary Table S3**. DEGs of annotated major cell types in spatial transcriptomic data.
**Supplementary Table S4**. DEGs of epithelial cell subclusters.
**Supplementary Table S5**. List of CancerSEA (http://biocc.hrbmu.edu.cn/CancerSEA/) tumor‐associated gene signatures.
**Supplementary Table S6**. DEGs of CAF cell subclusters.
**Supplementary Table S7**. DEGs of endothelial cell subclusters.
**Supplementary Table S8**. DEGs of macrophage cell subclusters.
**Supplementary Table S9**. List of macrophage‐related and metabolic gene signatures.
**Supplementary Table S10**. DEGs of DC cell subclusters.
**Supplementary Table S11**. DEGs of B/plasma cell subclusters.
**Supplementary Table S12**. Patient demographic data and tumor characteristics for each group.

## Data Availability

The raw data from the scRNA‐seq and spatial transcriptomic generated from OSCC patients have been deposited in the GSA (Genome Sequence Archive in BIG Data Center, Beijing Institute of Genomics, Chinese Academy of Sciences) database with accession number: PRJCA041463 (HRA011849 and HRA011848). The codes to process and analyze data are publicly available in the GitHub repository (https://github.com/dongwei1220/OSCC_betel‐nut_scRNA‐seq). Other relevant data are available from the corresponding authors upon reasonable request.
