# Supplementary material for: Comprehensive Integrated Single‐Cell and Spatial Transcriptomics Unveil the Dynamic Landscape of Betel Nut‐Associated Oral Mucosal Carcinogenesis and Its Tumor Microenvironment
Source: MedComm (2020). 2026 May 31;7(6):e70796. doi: 10.1002/mco2.70796 (PMC13239442; doi:10.1002/mco2.70796)
Supplement: Supplementary file 1 — Figure S1. Comprehensive single‐cell and spatial transcriptomic profiling of the TME in betel nut‐associated OSCC, related to Figure 1. (A–D) UMAP plots visualizing single‐cell transcriptomic data, colored by individual samples (A), sample group (B), sample type (C), and major cell types (D) in betel nut‐associated OSCC. Each dot represents an individual cell, and colors denote the individual samples, sample groups, sample type, and major cell types. (E) Bubble plots showing the relative average expression of canonical marker genes (x‐axis) across the cell clusters (y‐axis). Dot size indicates the proportion of cells expressing the gene, while color reflects the normalized expression level. (F) Stacked bar plots illustrating the distribution of major cell types between non‐betel nut and betel nut groups. Colors represent different cell types. (G, H) UMAP visualization of spatial transcriptomic spots from different samples (G) and cell clusters (H) in betel nut‐associated OSCC. Colors represent different samples and cell clusters. (I) Spatial plots depicting the distribution of cell clusters across different samples. Colors represent different cell clusters. Figure S2. Characterization of a highly invasive LAMC2+ malignant epithelial subpopulation in betel nut‐associated OSCC, related to Figure 2. (A) Bubble plots showing the relative average expression of top 5 marker genes (x‐axis) across the epithelial cell subclusters (y‐axis). Dot size indicates the proportion of cells expressing the gene, while color reflects the normalized expression level. (B) Stacked bar plots quantifying the relative abundance of 8 epithelial subclusters between non‐betel nut and betel nut groups. Colors represent different cell clusters. (C) Clustered heatmap of UCell signature scores across epithelial subclusters. Each row represents a tumor‐associated molecular signature (e.g., EMT, invasion, hypoxia), and each column represents an epithelial subcluster. (D) Pseudotime trajectory plot vi [file MCO2-7-e70796-s001.docx]

# Comprehensive Integrated Single-Cell and Spatial Transcriptomics Unveil the Dynamic Landscape of Betel Nut-Associated Oral Mucosal Carcinogenesis and Its Tumor Microenvironment

**Running title:** Single-Cell and Spatial Profiling of Betel Nut-Associated Oral Carcinogenesis

Wei Dong^1,2,#^, Shuojin Huang^1,#^, Yijun Wu^1^, Congyuan Cao^1^, Jiaxue Li^1^, Qianting He^1,*^, Anxun Wang^1,*^

^1^Department of Oral and Maxillofacial Surgery, The First Affiliated Hospital, Sun Yat-Sen University, Guangzhou, Guangdong, P. R. China.

^2^Guangzhou Women and Children’s Medical Center, Guangzhou Medical University, Guangzhou, Guangdong, P. R. China.

^#^These authors contributed equally to this work

*Correspondence: heqt3@mail.sysu.edu.cn (Q.T.H.), wanganx@mail.sysu.edu.cn (A.X.W.)

**Supplementary figure legends**


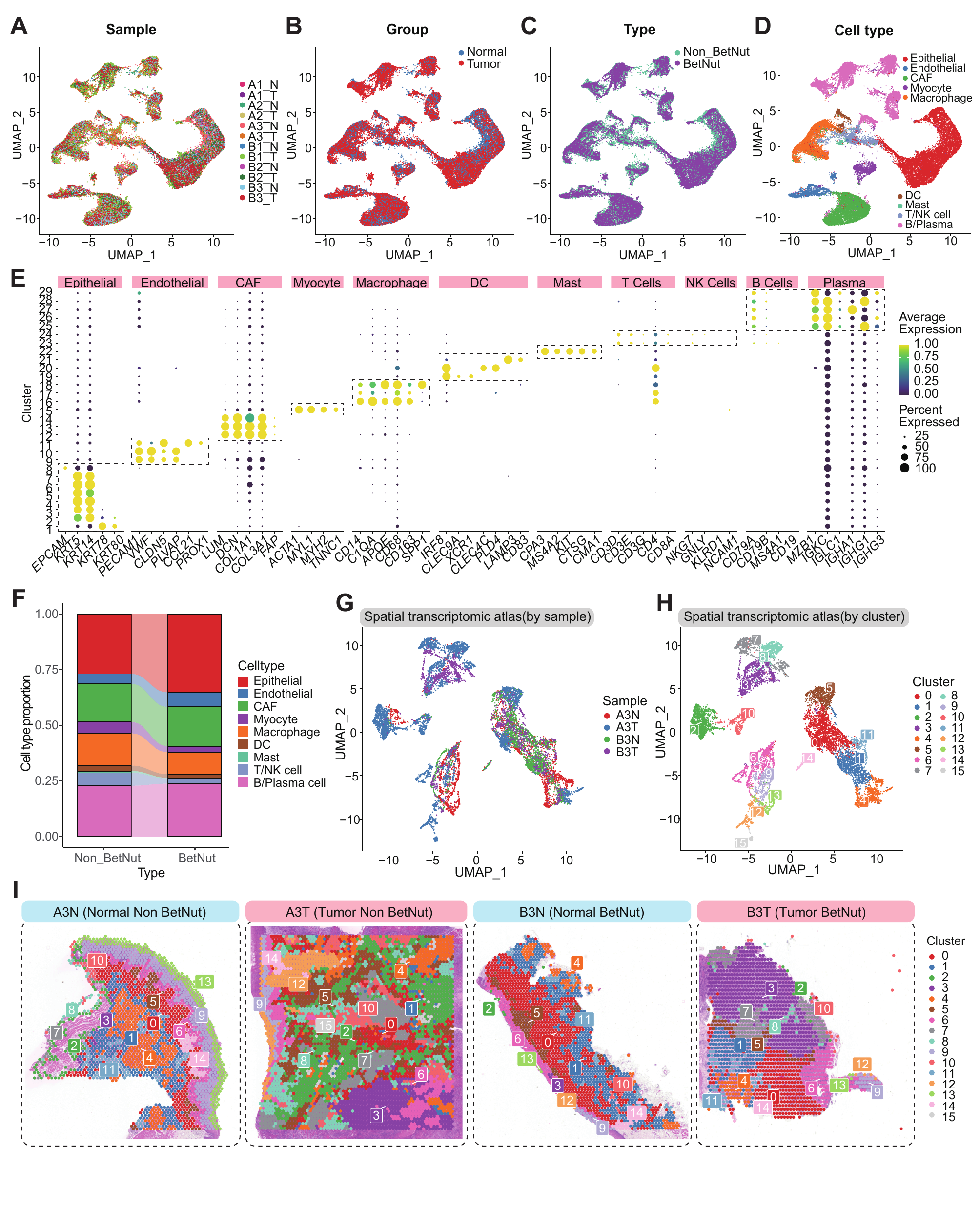


**Figure S1. Comprehensive single-cell and spatial transcriptomic profiling of the TME in betel nut-associated OSCC, related to Figure 1.**

**(A-D)** UMAP plots visualizing single-cell transcriptomic data, colored by individual samples (**A**), sample group (**B**), sample type (**C**), and major cell types (**D**) in betel nut-associated OSCC. Each dot represents an individual cell, and colors denote the individual samples, sample groups, sample type, and major cell types.

**(E)** Bubble plots showing the relative average expression of canonical marker genes (x-axis) across the cell clusters (y-axis). Dot size indicates the proportion of cells expressing the gene, while color reflects the normalized expression level.

**(F)** Stacked bar plots illustrating the distribution of major cell types between non-betel nut and betel nut groups. Colors represent different cell types.

**(G, H)** UMAP visualization of spatial transcriptomic spots from different samples (**G**) and cell clusters (**H**) in betel nut-associated OSCC. Colors represent different samples and cell clusters.

**(I)** Spatial plots depicting the distribution of cell clusters across different samples. Colors represent different cell clusters.


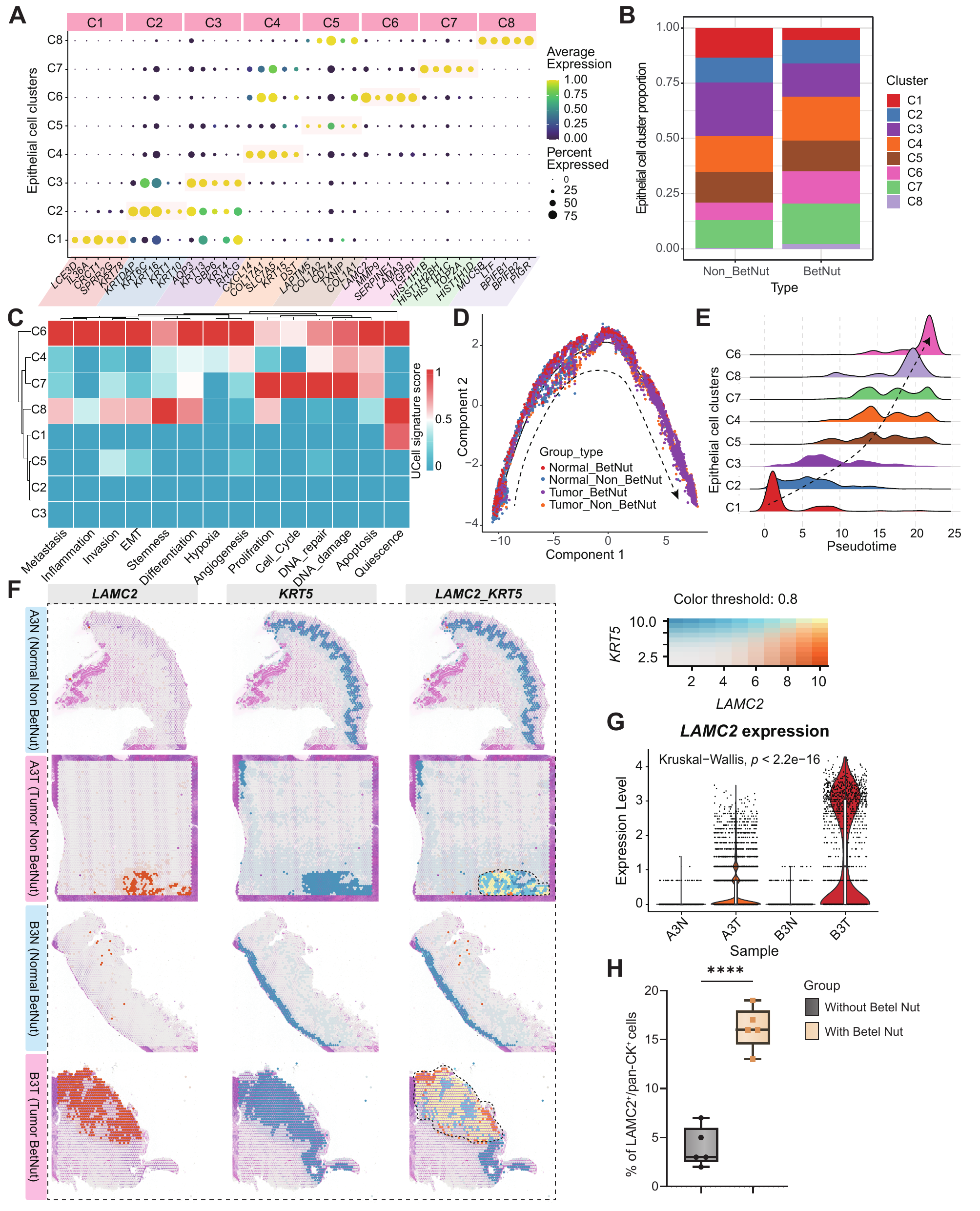


**Figure S2. Characterization of a highly invasive LAMC2^+^ malignant epithelial subpopulation in betel nut-associated OSCC, related to Figure 2.**

**(A)** Bubble plots showing the relative average expression of top 5 marker genes (x-axis) across the epithelial cell subclusters (y-axis). Dot size indicates the proportion of cells expressing the gene, while color reflects the normalized expression level.

**(B)** Stacked bar plots quantifying the relative abundance of 8 epithelial subclusters between non-betel nut and betel nut groups. Colors represent different cell clusters.

**(C)** Clustered heatmap of UCell signature scores across epithelial subclusters. Each row represents a tumor-associated molecular signature (e.g., EMT, invasion, hypoxia), and each column represents an epithelial subcluster.

**(D)** Pseudotime trajectory plot visualizing the inferred differentiation landscape of epithelial cells, colored by cell group types.

**(E)** Ridge plot showing the inferred epithelial cell differentiation trajectory among different cell subclusters. Colors represent different cell subclusters.

**(F)** Spatial transcriptomic plots illustrating the expression and co-expression patterns of *LAMC2* and *KRT5* genes in different samples, with LAMC2^+^ epithelial cells marked by irregular outlines.

**(G)** Violin plot showing the expression levels of *LAMC2* in different samples. Each dot represents an individual cell, and colors represent different samples.

**(H)** Box plot comparing the abundance of LAMC2*⁺* epithelial cells between betel nut chewers and non-chewers (n = 5, *****p* < 0.0001 by Student’s t-test).


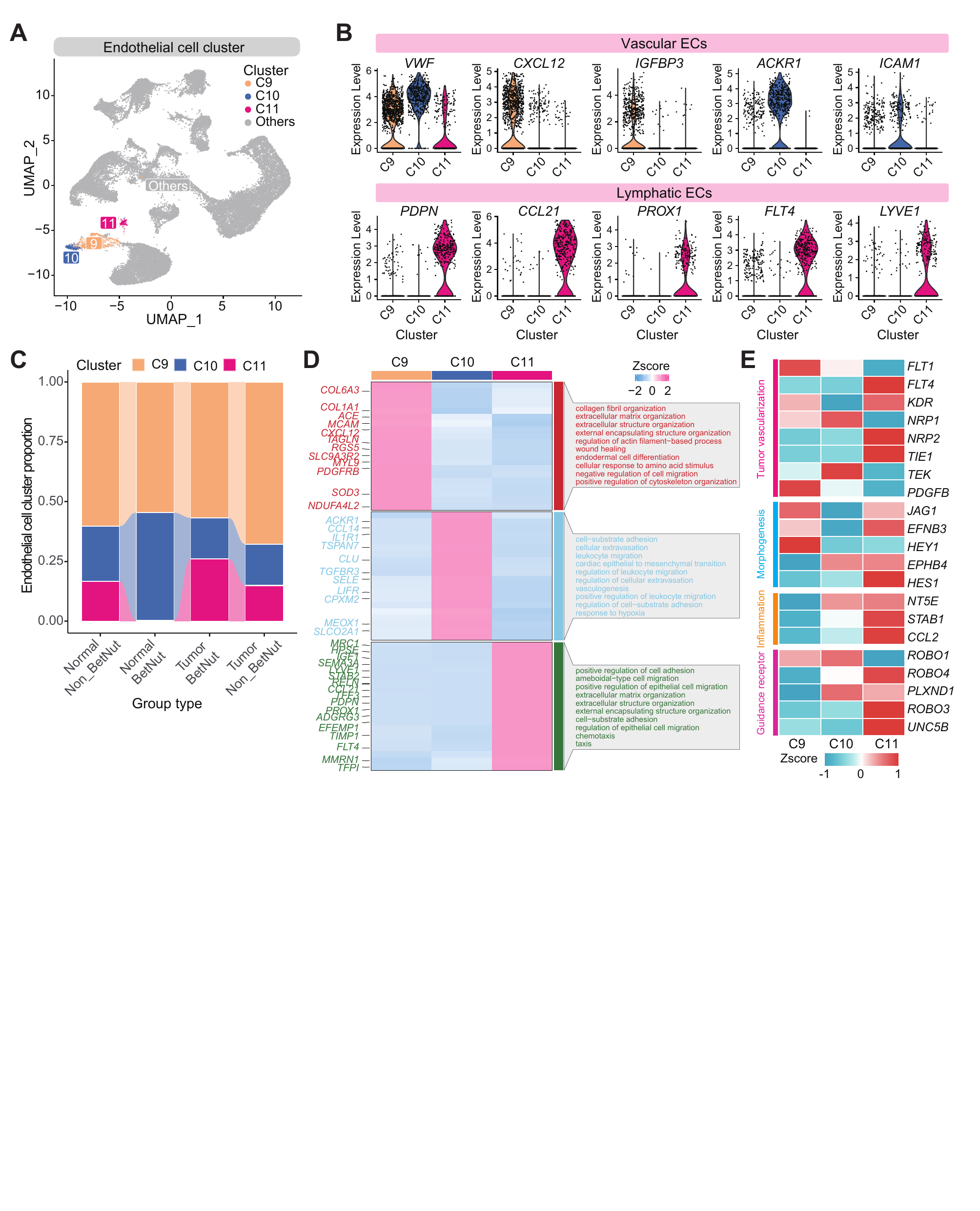


**Figure S3. Functional heterogeneity of endothelial cells in the TME of betel nut-associated oral cancer, related to Figure 3.**

**(A)** UMAP plot showing the three transcriptionally distinct endothelial cell subclusters. Colors represent different cell clusters.

**(B)** Violin plots comparing expression levels of key vascular endothelial and lymphatic endothelial markers across the three subclusters. Each dot represents an individual cell, and colors represent different cell subclusters.

**(C)** Stacked bar plots showing the proportional representation of endothelial subclusters across four different sample group types. Colors represent different cell subclusters.

**(D)** Heatmap of cell cluster-specific marker gene expression profiles with corresponding GO term enrichment (right).

**(E)** Heatmap depicting the expression of key genes associated with endothelial cell-associated functional programs across three endothelial subclusters.


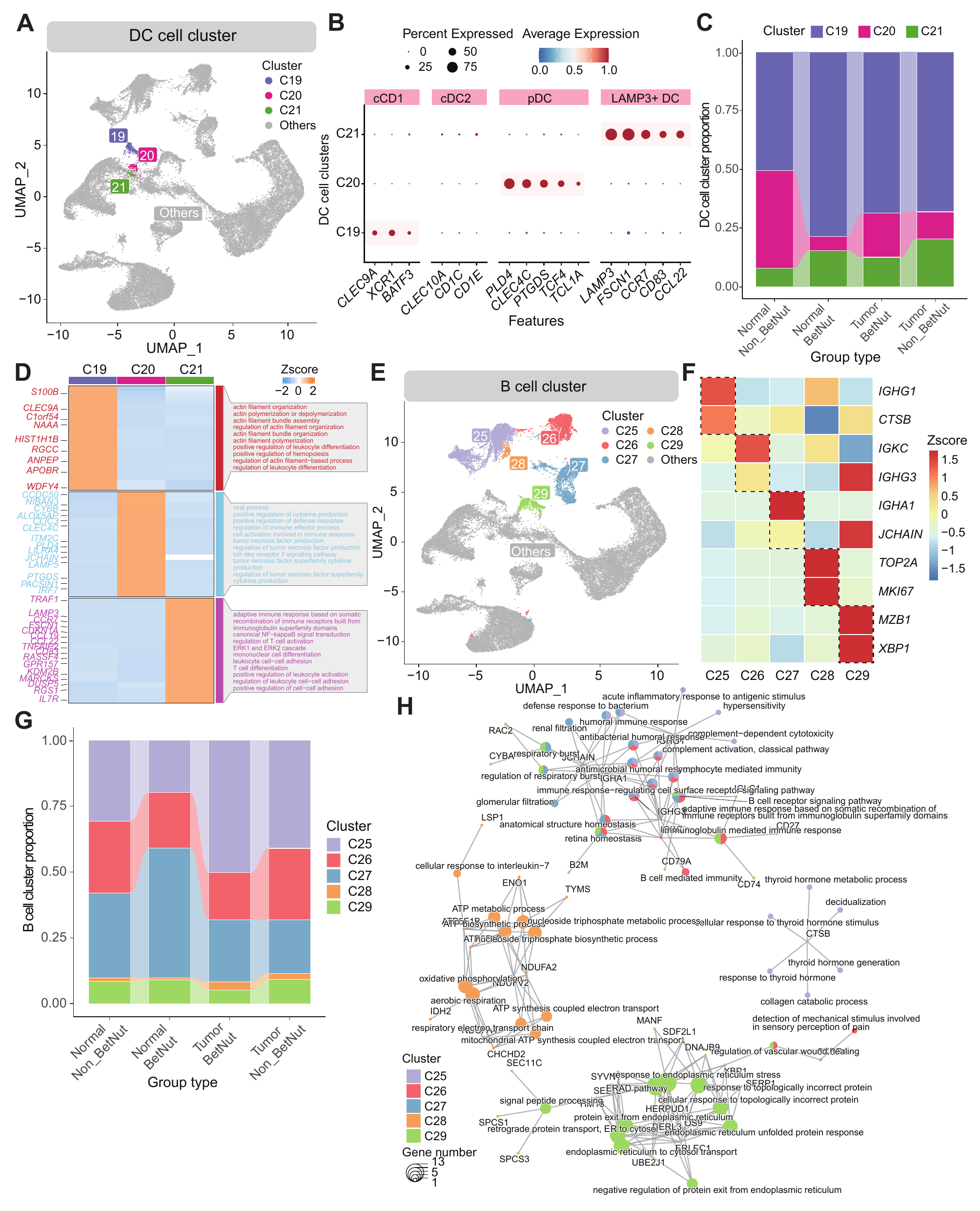


**Figure S4. Profiling immune cell heterogeneity and functional diversity in the TME of betel nut-associated OSCC, related to Figure 4.**

**(A)** UMAP plot showing the three transcriptionally distinct DC cell subclusters in betel nut-associated OSCC. Colors represent different cell clusters.

**(B)** Bubble plots showing the relative average expression of canonical marker genes (x-axis) across the DC cell subclusters (y-axis). Dot size indicates the proportion of cells expressing the gene, while color reflects the normalized expression level.

**(C)** Stacked bar plot displaying the distribution of DC subclusters across four different sample group types. Colors represent different cell subclusters.

**(D)** Heatmap of DC cell cluster-specific marker gene expression profiles, annotated with enriched GO terms (right).

**(E)** UMAP plot showing the five B/Plasma cell subclusters in betel nut-associated OSCC. Colors represent different cell clusters.

**(F)** Heatmap displaying the average expression profiles of marker genes across five B/Plasma cell subclusters.

**(G)** Stacked bar plot showing proportion of B/Plasma cell subtypes across four different sample group types. Colors represent different cell subclusters.

**(H)** Functional enrichment network of GO terms and associated genes across different B/Plasma cell subclusters. Colors represent different cell clusters.


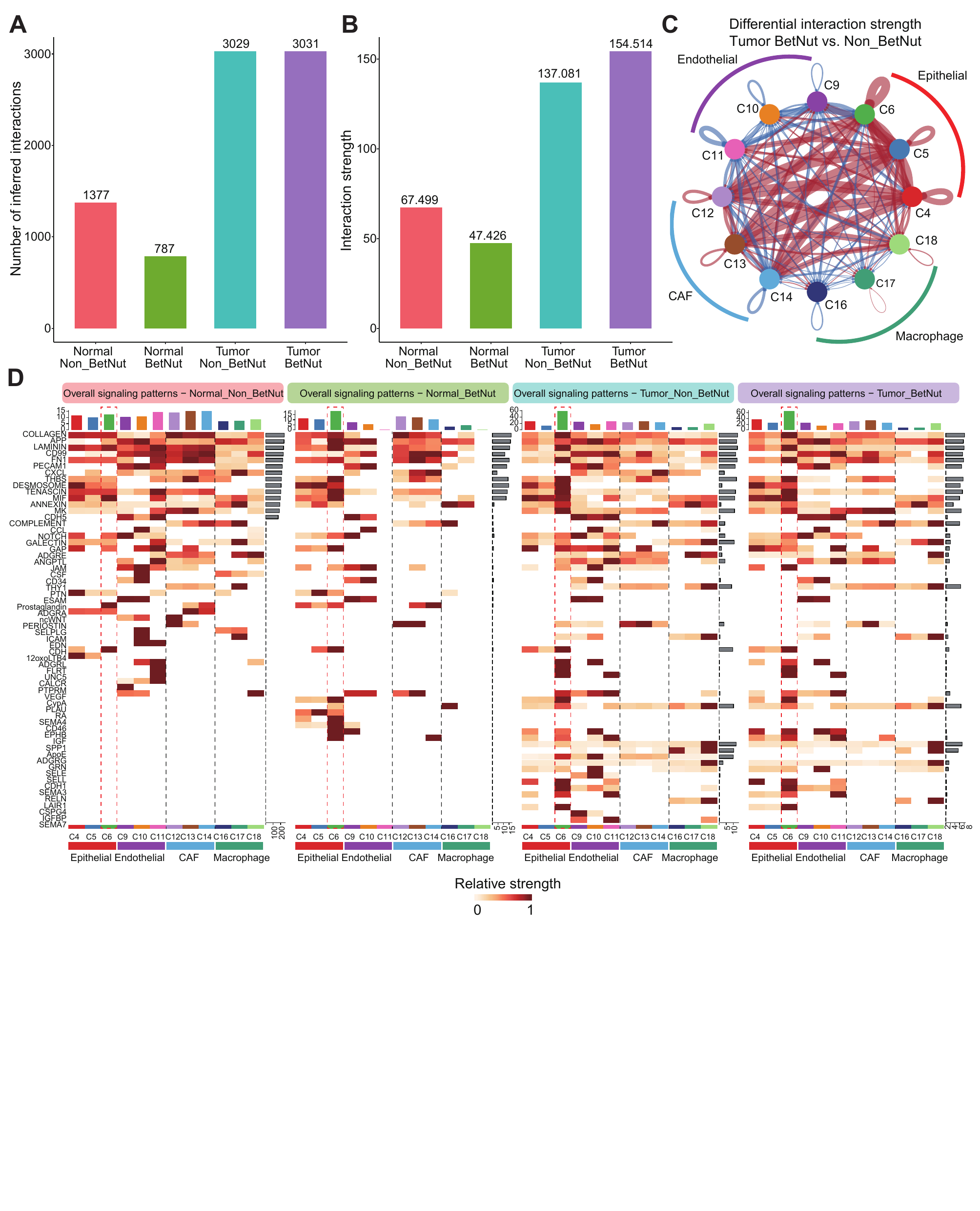


**Figure S5. Malignant epithelium-CAF crosstalk drives tumor progression in betel nut-associated OSCC, related to Figure 5.**

**(A, B)** Bar plots quantifying the total number of inferred ligand-receptor interactions (**A**) and the overall interaction strength (**B**) among TME cell types in betel nut-associated OSCC.

**(C)** Circular interaction network comparing differential intercellular communication between betel nut-associated and non-betel nut-associated OSCC among epithelial cells, endothelial cells, CAFs, and macrophages. Node colors represent cell subclusters, and edge colors denote differential interaction strength, with red representing increased interaction strength in betel nut tumor samples, blue represents decreased interaction strength in betel nut tumor samples.

**(D)** Heatmap summarizing the strength of overall signaling pathways among different cell clusters in four different sample group types. Each row corresponds to a specific signaling pathway, and columns represent recipient clusters. Cell type identities are annotated below.

**Supplementary tables**

**Supplementary Table S1. Detailed baseline information of patients included in scRNA sequencing and spatial transcriptomic.**

**Supplementary Table S2. Differential expression genes (DEGs) of 9 major cell types in scRNA-seq data.**

**Supplementary Table S3. DEGs of annotated major cell types in spatial transcriptomic data.**

**Supplementary Table S4. DEGs of epithelial cell subclusters.**

**Supplementary Table S5. List of CancerSEA (http://biocc.hrbmu.edu.cn/CancerSEA/) tumor-associated gene signatures.**

**Supplementary Table S6. DEGs of CAF cell subclusters.**

**Supplementary Table S7. DEGs of endothelial cell subclusters.**

**Supplementary Table S8. DEGs of macrophage cell subclusters.**

**Supplementary Table S9. List of macrophage-related and metabolic gene signatures.**

**Supplementary Table S10. DEGs of DC cell subclusters.**

**Supplementary Table S11. DEGs of B/Plasma cell subclusters.**

**Supplementary Table S12. Patient demographic data and tumor characteristics for each group.**
